# *MeGATA*s, functional generalists in interactions between cassava growth and development, and abiotic stresses

**DOI:** 10.1093/aobpla/plac057

**Published:** 2022-11-25

**Authors:** Yan-Liu Wu, Yu-Lan Chen, Li Wei, Xian-Wei Fan, Ming-You Dong, You-Zhi Li

**Affiliations:** State Key Laboratory for Conservation and Utilization of Subtropical Agro-bioresources/College of Life Science and Technology, Guangxi University, 100 Daxue Road, Nanning, Guangxi 530004, P.R. China; State Key Laboratory for Conservation and Utilization of Subtropical Agro-bioresources/College of Life Science and Technology, Guangxi University, 100 Daxue Road, Nanning, Guangxi 530004, P.R. China; State Key Laboratory for Conservation and Utilization of Subtropical Agro-bioresources/College of Life Science and Technology, Guangxi University, 100 Daxue Road, Nanning, Guangxi 530004, P.R. China; State Key Laboratory for Conservation and Utilization of Subtropical Agro-bioresources/College of Life Science and Technology, Guangxi University, 100 Daxue Road, Nanning, Guangxi 530004, P.R. China; State Key Laboratory for Conservation and Utilization of Subtropical Agro-bioresources/College of Life Science and Technology, Guangxi University, 100 Daxue Road, Nanning, Guangxi 530004, P.R. China; State Key Laboratory for Conservation and Utilization of Subtropical Agro-bioresources/College of Life Science and Technology, Guangxi University, 100 Daxue Road, Nanning, Guangxi 530004, P.R. China

**Keywords:** Cassava, environmental response, functional evolution, GATA domain-containing genes, gene duplication, subcellular localization

## Abstract

The proteins with DNA-binding preference to the consensus DNA sequence (A/T) GATA (A/G) belong to a GATA transcription factor family, with a wide array of biological processes in plants. Cassava (*Manihot esculenta*) is an important food crop with high production of starch in storage roots. Little was however known about cassava GATA domain-containing genes (*MeGATA*s). Thirty-six *MeGATA*s, *MeGATA1* to *MeGATA36*, were found in this study. Some *MeGATA*s showed a collinear relationship with orthologous genes of *Arabidopsis*, poplar and potato, rice, maize and sorghum. Eight *MeGATA*-encoded proteins (MeGATAs) analysed were all localized in the nucleus. Some MeGATAs had potentials of binding ligands and/or enzyme activity. One pair of tandem-duplicated *MeGATA17–MeGATA18* and 30 pairs of whole genome-duplicated *MeGATA*s were found. Fourteen *MeGATA*s showed low or no expression in the tissues. Nine analysed *MeGATA*s showed expression responses to abiotic stresses and exogenous phytohormones. Three groups of MeGATA protein interactions were found. Fifty-three miRNAs which can target 18 *MeGATA*s were identified. Eight MeGATAs were found to target other 292 cassava genes, which were directed to radial pattern formation and phyllome development by gene ontology enrichment, and autophagy by Kyoto Encyclopaedia of Genes and Genomes enrichment. These data suggest that *MeGATA*s are functional generalists in interactions between cassava growth and development, abiotic stresses and starch metabolism.

## Teaser

Cassava *MeGATAs*play multiple roles in growth and development, abiotic stresses and starch metabolism of cassava.

## Introduction

The proteins with DNA-binding preference to the consensus DNA sequence (A/T) GATA (A/G) belong to GATA transcription factors (TFs), have evolutionarily blossomed into a GATA family (Patient and McGhee 2002), and have been found throughout eukaryotes from fungi (Scazzocchio 2000), plants (Behringer and Schwechheimer 2015) and invertebrates (Patient and McGhee 2002) to vertebrates (He *et al.* 2007). GATA proteins can interact with the WGATAR (W = T or A; R = G or A) sequence motifs in eukaryotes (An *et al.* 2020). It is reported that GATA proteins are associated with biological processes including growth and development such as seed germination, chloroplast development, flower development, light response, and lateral root initiation identity, nitrogen metabolism, and photosynthetic electron transfer and carbon assimilation in the leaf, cell division, carbohydrate utilization in the stem, and nitrogen uptake in the root of *Populus trichocarpa* (An *et al.* 2020), as well as responses to abiotic stresses (Scazzocchio 2000; Patient and McGhee 2002; He *et al.* 2007; Behringer and Schwechheimer 2015; Block and Shapira 2015; Gupta *et al.* 2017; An *et al.* 2020). The GATA family is comparatively larger in plants relative to animals (Behringer and Schwechheimer 2015). In plants, the interest in GATA proteins is that GATA motifs are enriched in promoters of light-regulated genes and circadian clock-controlled genes (Behringer and Schwechheimer 2015). In fact, in plants, more knowledge of GATA TFs learns from *Arabidopsis* and rice (Reyes *et al.* 2004). It has been found that C-terminal leucine–leucine–methionine (LLM) domain-containing B-GATAs from *Arabidopsis* control seed germination, greening, senescence and flowering time downstream from several growth regulatory signals (Behringer and Schwechheimer 2015). The expression regulation of some genes of the GATA TF family is very complex, not only by numerous other TFs but also by themselves such as GATA-1 gene (Morceau *et al.* 2004). GATA TFs can also affect the expression of other genes via interplay with chromatin domain (Bresnick *et al.* 2005) and have been considered as tissue-specific master regulators for induced responses (Block and Shapira 2015). The C-terminal LLM domain is seemingly specific for the *Brassicaceae* family, and an N-terminal HANABA TARANU domain has species specificity in monocots such as rice, maize and barley (Behringer and Schwechheimer 2015). In plants, some GATA TFs even have a unique degenerate LLM or HAN domain (Behringer and Schwechheimer 2015).

Cassava (*Manihot esculenta*) is an important food crop with high production of starch in storage roots in Africa, Asia, Latin America and the Caribbean (El-Sharkawy 2004; Okogbenin *et al.* 2013). Cassava can be produced adequately in drought conditions in marginal environments of the farmland and the barren slopes; however, the complex traits of growth, development and multiple botanical aspects (Caltayud *et al.* 2002; El-Sharkawy 2004; Nassar *et al.* 2008; Okogbenin *et al.* 2013) make the many mechanisms a mystery.

The purpose of this study was to investigate GATA family genes of cassava, *MeGATA*s, in order to provide some clues for further analysis of their functions in cassava growth and development.

## Materials and Methods

### Identification and general characterization analysis of MeGATA proteins

The first step to identify putative GATA motif-containing proteins of cassava was to search the protein data set of cassava in v9.0 phytozome database (https://phytozome.jgi.doe.gov/) by using the hidden Markov model (HMM) sequence (PF00320) of the GATA family proteins and the HMMER3 tool under 1E-value of <0.01 (Eddy 2011). In order to prevent loss of some candidate cassava GATA proteins, the second step was to conduct BLASTP analysis between those candidate cassava GATA proteins and *Arabidopsis* GATA proteins and rice GATA proteins from the uniprot database (https://www.uniprot.org/) under 1E-value of <0.01. Finally, candidate GATA proteins of cassava were further confirmed by using the CDD tool under a threshold value of 0.01 and maximum hits of 500 (Marchler-Bauer *et al.* 2017) as well as Pfam tool under 1E-value of <0.05 (Finn *et al.* 2016; https://pfam.xfam.org/).

The molecular weights and isoelectric points of the proteins were analysed by using the ExPASy tool (http://www.expasy.org/tools/). Multiple homology alignment of the proteins was conducted by using the Clustal X 2.0 tool (Larkin *et al.* 2007). The phylogenetic tree of the proteins was constructed using the maximum likelihood method and Jones Taylor Thornton amino acid substitution model by using the MEGA7 tool (Kumar *et al.* 2016; www.megasoftware.net) under 1000 bootstrap replications. The subcellular localization of the proteins was predicted by using the online cello tool (Yu *et al.* 2006). The conservative sequence motifs of the proteins were analysed by using the MEME tools (Bailey *et al.* 2015) and then functionally annotated by searching the InterProScan database (http://www.ebi.ac.uk/Tools/pfa/iprscan/) (Jones *et al*. 2014).

The potential enzymes and ligands and ligand-binding sites were predicted through the online I-TASSER server (http://zhanglab.ccmb.med.umich.edu/I-TASSER) under built-in default values (Zhang and Skolnick 2004; Zhang 2008; Roy *et al.* 2010; Yang *et al.* 2015), where the server matched the predicted 3D models to the proteins from three independent libraries containing proteins of known enzyme classification (EC) number, gene ontology (GO) vocabulary, and ligand-binding sites from BioLiP database. Predictions of enzymes and ligands were then based on amino acid sequences of the proteins under a confidence score (C-score) ranging from 0 to 1, which was calculated based on the significance of threading template alignments and the convergence parameters of the structure assembly simulations, where a higher score indicates a more reliable prediction.

### Analysis of DNA structure and motifs of *MeGATA*s

The *MeGATA* DNA structure was analysed by using the TBtools (Chen *et al.* 2020; https://github.com/CJ-Chen/TBtools). The motif sequences of *MeGATAs* were identified by using the MEME tools (Bailey *et al.* 2015; https://meme-suite.org/meme/tools/meme) and annotated through the InterProScan database (Quevillon *et al.* 2005; http://www.ebi.ac.uk/Tools/pfa/iprscan/).

### Chromosome localization and collinearity analysis of *MeGATA*s

Chromosome localization of *MeGATA*s was conducted by using the Circos tool (Gu *et al.* 2014). The collinearity of *MeGATA*s was analysed by using the DualSystenyPlotter software in the TBtools (Chen *et al.* 2020; https://github.com/CJ-Chen/TBtools). The non-synonymous substitution rate (*K*_a_) and synonymous substitution rate (*K*_s_) of *MeGATA*s in the gene collinearity were calculated by using the ParaAT tool (Zhang *et al.* 2012). The *K*_a_/*K*_s_ values were calculated by using the_Calculator 2.0 software (Wang *et al.* 2010). The gene duplicate time was estimated based on *K*_s_/2*λ* (Koch *et al.* 2000), where *λ* = 1.5 × 10^−8^.

### Prediction of *cis*-acting elements in promoters of *MeGATA*s

The candidate promoter region was assumed to be localized in 1500-bp genomic DNA segments upstream of the start codons of *MeGATA*s. The *cis*-acting elements were analysed by using the NewPLACE tool (Higo *et al.* 1999; https://sogo.dna.affrc.go.jp/cgi-bin/sogo.cgi?lang=en&pj=640&action=page&page=newplace).

### Prediction of interactions of MeGATAs–MeGATAs and MeGATAs–other cassava proteins

The protein interactions were based on the Search Tool for the Retrieval of Interacting Genes (https://string-db.org/). Briefly, in the ‘Search’ window, the ‘Multiple proteins’ followed by the ID number of MeGATAs such as cassava 4.1_033370m were selected. In the ‘Basic Setting’ window, the ‘Network type’ selected was ‘full STRING network’, and all items under ‘active interaction sources’ were selected. A low confidence (0.15) was used as the ‘minimum required interaction score’.

### Prediction of downstream target genes regulated by MeGATAs

The target genes regulated by MeGATAs were predicted by using the online PlantRegMap tool with a version 5 Plant Transcription Factor Database (http://plantregmap.gao-lab.org/network.php) (Tian *et al.* 2020).

In this study, this tool was operated under the specified inputs of species *M*. *esculenta*, all organs (inflorescence, root, root non-hair, seed and seedling), FunTFBS (TF binding sites) method, TF (retrieve targets) mode, and cassava *MeGATA* ID. The downstream target genes by MeGATAs were identified and retrieved when the correlation test was significant (*P* €≦ 0.05) with a correlation score higher than 0.5.

The GO analysis was conducted to analyse potential functions of the related genes at a *P*. adjust value of <0.05. The KEGG analysis was conducted to analyse potential metabolic pathways of the related genes at a *P*-value of <0.05 in the online Omicshare tool (https://www.omicshare.com/tools/Home/Soft/getsoft).

### Prediction of microRNA–*MeGATA* regulatory networks

The (miRNA)–*MeGATA* regulatory networks were predicted according to the previous methods described by Su *et al.* (2021) but with some modifications. In brief, the *MeGATA*-targeting miRNAs were predicted with *MeGATA*s’ coding sequences (CDS) by using the psRNATarget server (http://plantgrn.noble.org/psRNATarget/home) under default parameters except that a maximum expectation was 5.0. The miRNA-targeted sites were those highly complementary to *MeGATA*s’ CDSs. The interaction networks were created by using the Cytoscape V3.8.2 software (https://cytoscape.org/download.html).

### Analysis of expression profile of *MeGATA*s in cassava tissues

The expression analysis of *MeGATA*s was based on the transcriptome data sets in the RNA-seq read archives of cassava (**see****Supporting Information—Table S1**; Wang *et al.* 2014), which were involved in early storage roots 75 d after planting (DAP), medium tuber roots (120 DAP), late storage roots (150 DAP), stems (90 DAP) and leaves (90 DAP) for Arg7; and early storage roots (75 DAP), medium tuber roots (120 DAP), late storage roots (150 DAP) and leaves (90 DAP) for KU50; and roots (90 DAP), stems (90 DAP) and leaves (90 DAP) for W14. The expression levels of *MeGATA*s were estimated on the basis of the log_2_ of Fragments Per Kilobase of transcript per Million-fragments mapped values of gene expression in the data sets.

### RNA isolation and the first-strand cDNA synthesis

The total RNA was isolated from 100 mg of cassava leaves by using the OmniPlant RNA Kit (DNase I) (ComWin Biotech Co., Ltd, China). For isolated RNA, the quality was controlled through agarose gel electrophoresis and by using the NanoDrop 2000 (Thermo, Waltham, MA, USA), and the concentration was determined by using the NanoDrop 2000. The first-strand cDNA synthesis was conducted with 1 μg of quality-controlled RNA by using the PrimeScript^™^ RT reagent Kit with gDNA Eraser [TaKaRa Biomedical Technology (Beijing), China]. Then, the synthesized cDNA product was diluted 10 times with RNA-free water for further use.

### Analysis of subcellular localization of MeGATAs

First, CDS DNA of *MeGATA*s was synthesized by PCR with the first-strand cDNA as template and sequence-specific primers **[see****Supporting Information—Table S2****]** by using the 2× PrimeSTAR Max Premix kit [TaKaRa Biomedical Technology (Beijing), China]. In brief, DNA of CDS (without stop code) of *MeGATA*s was ligated into plasmid pCambia2300-35S-eGFP collected by our laboratory to generate pCambia2300-35S-*MeGATA*s-GFP. The primers used for construction were listed in **Supporting Information—Table S3**. Rice protoplast preparation as well as protoplast co-transformation of both pCambia2300-35S-*MeGATA*s-GFP and nuclear localization marker plasmid pA7-Ghd7-mCherry (Xue *et al.* 2008) were carried out as previously described by Yang *et al.* (2014). The subcellular localization was evaluated by a Leica TCS SP8 laser scanning confocal microscope (Germany), where the excitation wavelengths used were 488 nm for GFP within a fluorescence acquisition band range from 501 to 520 nm, and 552 nm for mCherry within a fluorescence acquisition band range from 590 to 620 nm.

### Treatments of pot-grown cassava

The stem cuttings with three buds from plants of cassava South China 124 (SC124) field-grown for 180 d were planted into pots containing perlite which was saturated with water before potting and then grew for 42 d in the growth chamber with 16-h light/8-h dark and 55% air humidity. The cassava plantlets were then treated by high temperature at 42 °C while other plantlets were cultured under normal temperature at 27 °C as the control. The cassava plantlets were treated by natural drought without watering while other plantlets were normally irrigated with tap water at 27 °C as the control. The plants were with 200 mM NaCl while other plantlets were normally irrigated with tap water at 27 °C as the control. The roots of cassava plantlets were soaked in 100 µM abscisic acid (ABA), 100 µM indole 3-acetic acid (IAA) and 100 µM salicylic acid (SA), respectively, while the leaves were sprayed with 100 µM of ABA, IAA or SA. The plantlets concurrently soaked in and sprayed with tap water at 27 °C were used as corresponding controls, respectively.

### Analysis of *MeGATA* expression in pot-grown cassava SC124 by real-time quantitative reverse transcriptase-PCR (RT-PCR)

The RT-qPCR was performed by using the StepOne™ Real-Time PCR System (Thermo Fisher Scientific, USA) and conducted in a 20-µL reaction system containing the 10 fold diluted first-strand cDNA solution and sequence-specific primers **[see****Supporting Information—Table S4****]** by using the ChamQ™ Universal SYBR qPCR Master Mix kit (Vazyme, China). The internal control gene was Cassava4.1_006776 (Hu *et al.* 2016). The specificity of sequence primers was determined based on the cassava data set (taxid:3983) by using the primer-blast tool in the NCBI (https://www.ncbi.nlm.nih.gov/tools/primer-blast/index.cgi?LINK_LOC=BlastHome) under the default parameters. The relative expression level of *MeGATA*s between treatment and control was calculated following the 2^−ΔΔCT^ method (Schmittgen and Livak 2008), where ΔΔCt = [(Ct_*MeGATA*_ – Ct_Cassava4.1_006776_) under treatment] – [(Ct_*MeGATA*_ – Ct_Cassava4.1_006776_) under control]. The three biological replicates for each gene were conducted with leaves of three individual plants. Differential expression of the genes between the treatments was defined at a significance level of *P* < 0.05 based on Duncan’s multiple range test.

### Statistical analysis

The statistical package for SPSS 18 program was used for statistical analysis. One-way ANOVA was performed to evaluate significant differences between data at *P* < 0.05.

## Results

### Cassava MeGATA proteins and *MeGATA* genes

A total of 36 MeGATAs, MeGATA1 to MeGATA36, were identified, which ranged in length from 106 amino acids in MeGATA21 to 544 amino acids in MeGATA1, in isoelectric point from 4.73 in MeGATA17 to 11.02 in MeGATA21 and in molecular weight from 12170.4 Da in MeGATA21 to 60489.67 Da in MeGATA (Table 1). On the whole, MeGATAs were predicted to be localized in the nucleus (Table 1) and had a total of 20 conservative motif sequences **[see****Supporting Information—Table S5****]**. Some MeGATAs were predicted to have potential ligand binding or enzyme activity, or both (Table 2).

**Table 1.** Basic information on *MeGATA*s and MeGATAs.

	MeGATA					MeGATA				
Name	Locus ID*	ID number of the corresponding *Arabidopsis* homologue	Transcript			Amino acid residue number	Subcellular localization		Molecular weight (Da)	Isoelectric point
			Number	Intron	Exon		Predicated	Identified by this study		
*MeGATA1*	Manes.01G087500	At4g17570	1	7	8	544	Nucleus	/	60 489.67	6.58
*MeGATA2*	Manes.01G135900	At3g50870	1	1	2	248	Nucleus	/	27 743.64	8.51
*MeGATA3*	Manes.01G224400	At5g49300	1	2	3	135	Nucleus	/	14 994.52	9.69
*MeGATA4*	Manes.02G044400	At4g17570	1	7	8	542	Nucleus	/	60 031.15	6.55
*MeGATA5*	Manes.02G094300	At3g50870	1	1	2	217	Nucleus	/	24 098.83	9.21
*MeGATA6*	Manes.02G099500	At5g66320	1	2	3	368	Nucleus	Nucleus	39 962.68	6.04
*MeGATA7*	Manes.03G033200	At5g56860	1	2	3	304	Nucleus	Nucleus	33 924.06	8.96
*MeGATA8*	Manes.03G047800	At4g26150	1	2	3	297	Nucleus	Nucleus	32 950.03	8.72
*MeGATA9*	Manes.03G059100	At5g25830	1	1	2	362	Nucleus	/	39 921.81	5.88
*MeGATA10*	Manes.03G154500	At3g24050	4	2	3	261	Nucleus	/	28 957.71	7.62
*MeGATA11*	Manes.03G201300	At3g06740	1	2	3	143	Nucleus	/	15 599.58	9.76
*MeGATA12*	Manes.04G084400	At5g66320	2	1	2	335	Nucleus	Nucleus	37 463.97	4.95
*MeGATA13*	Manes.04G132800	At4g32890	1	1	2	325	Nucleus	/	36 354.3	6.34
*MeGATA14*	Manes.05G050300	At2g45050	1	1	2	263	Nucleus	/	29 437.87	7.21
*MeGATA15*	Manes.05G189500	At3g21175	2	9	10	355	Nucleus	/	38 965.4	4.79
*MeGATA16*	Manes.05G189600	At4g24470	1	6	7	285	Nucleus	/	31 044.27	6.45
*MeGATA17*	Manes.07G041200	At3g21175	2	9	10	364	Nucleus	//	40 162.47	4.73
*MeGATA18*	Manes.07G041300	At1g51600	2	6	7	296	Nucleus	/	31 399.92	5.24
*MeGATA19*	Manes.07G076400	At4g17570	1	2	3		Nucleus	/	29 659.47	8.36
*MeGATA20*	Manes.07G099600	At4g32890	1	3	4		Nucleus	/	38 318.79	6.01
*MeGATA21*	Manes.08G113300	At3g06740	1	1	2		Nucleus	/	12 170.4	11.02
*MeGATA22*	Manes.08G149300	At5g25830	1	1	2		Nucleus	/	33 593.55	6.46
*MeGATA23*	Manes.09G142600	At4g32890	3	1	2		Nucleus	/	32 548.46	8.37
*MeGATA24*	Manes.09G174900	At3g06740	1	2	3		Nucleus	Nucleus	17 653.33	9.66
*MeGATA25*	Manes.10G046800	At4g32890	1	3	4		Nucleus	/	38 570.44	6.66
*MeGATA26*	Manes.10G097400	At3g21175	2	6	7		Nucleus	/	32 210.87	5.28
*MeGATA27*	Manes.11G034900	At4g32890	1	1	2		Nucleus	/	36 000.67	6.37
*MeGATA28*	Manes.11G146600	At1g08010	1	2	3		Nucleus	/	35 426.06	8.65
*MeGATA29*	Manes.15G007100	At3g06740	1	2	3		Nucleus	/	15 546.53	9.79
*MeGATA30*	Manes.15G049400	At3g24050	1	1	2		Nucleus	/	29 578.39	7
*MeGATA31*	Manes.15G103300	At3g20750	1	1	2		Nucleus	/	15 975.67	9.3
*MeGATA32*	Manes.16G074900	At5g25830	1	1	2		Nucleus	/	40 216.31	6
*MeGATA33*	Manes.16G080400	At5g56860	1	2	3		Nucleus	Nucleus	33 804.27	9.16
*MeGATA34*	Manes.16G102600	At5g56860	1	2	3		Nucleus	Nucleus	33 672.75	8.95
*MeGATA35*	Manes.18G056300	At4g24470	1	6	7		Nucleus	/	30 486.6	6.89
*MeGATA36*	Manes.18G056400	At3g21175	1	9	10		Nucleus	Nucleus	33 558.36	5.63

/, not identified.

*In https://phytozome.jgi.doe.gov/.

**Table 2.** Potential of ligand binding and enzyme activity predicted in MeGATAs.

Evolutional group on the phylogenetic tree	*MeGATA*	Ligand*			Enzyme activity			
		Name	Amino acid residue at binding site	C-score	Name	Enzyme entry	Amino acid residue at active site	C-score^EC^
Group I	MeGATA6	S-[1-OXLY-2,2,5,5,-Teramethyl-2,5-Dihydro-1H-pyrrrol-3-yl)] methanesulfonothioate	Cys 285; Ser 286	0.07	NA			
	MeGATA9	*Cis*-diammine(pyridine) chloroplatinum(II)	Tyr 282; Val 280; Gly 279	0.17	Non-specific serine/threonine protein kinase	EC 2.7.11.1	Asp 249	0.134
		PEPTIDE	Arg 286; Val 280; Tyr 282; Lys 283	0.10				
	MeGATA10	Arginine	Gly 186; Ala 187; Lys 189; Pro 197	0.09	Type II site-specific deoxyribonuclease	EC 3.1.21.4	Lys 78	0.068
		Oligomycine B	Gly 47; Ala 51; Phe 55	0.07				
		Mg^2+^	Asp 17; Glu 242	0.07				
	MeGATA12	Zn^2+^	Cys 257; Cys 260	0.12	DNA helicases	EC 3.6.4.12	Lys 287	0.083
	MeGATA13	Zn^2+^	Cys 220; His 222; Cys 223; Ala 244	0.20	NA			
	MeGATA14	Zn^2+^	Cys 169; Cys 172; Cys 191; Cys 194	0.18	NA			
		Mg^2+^	Val 196; Lys 199	0.07				
	MeGATA20	8k6/BioLip ID BL0234940	Arg 269; Ala 327	0.08	Non-specific serine/threonine protein kinase	EC 2.7.11.1	Lys 276	0.129
		Zn^2+^	Tyr 286; Glu 294	0.06				
	MeGATA22	Ca^2+^	Asn 253; Ala 254	0.12	NA			
		Peptide	Asn 253; Gly 265	0.07				
	MeGATA23	Chlorophyll a	Ala 244; Cys 245	0.15	3’,5’-cyclic-nucleotide phosphodiesterase	EC 3.1.4.17	Gly 246	0.105
		7-[2-(5-methyl-1-phenyl-1H-benzimidazol-2-YL) ethyl]imidazo [1,5-B] pyridazine	Gln 230; Tyr 249; Lys 250; Gly 252; Arg 253	0.12				
		T0M	His 222; Cys 223; Gln 226	0.09				
	MeGATA25	Zn^2+^	Cys 278, Cys 281	0.29	Non-specific serine/threonine protein kinase	EC 2.7.11.1	Lys 255; Il 261; Gly 319	0.128
	MeGATA27	Zn^2+^	Cys 220; Cys 223; Cys 242; Cys 245	0.31	NA			
		Alpha-L-fucopyranose	Val 247; Lys 250; Ser 251; Ser 272	0.13				
		Mg^2+^	Asn 30; Ser 34; Arg 39	0.12				
	MeGATA28	Chlorophyll a	Trp 239; Cys 254	0.08	NA			
		Maltotetraose	Leu 207; Lys 227; Arg 258	0.08				
		Sulfate (SO_4_^2−^)	Arg 240; Gly 254; Arg 256; Tyr 257					
	MeGATA30	Ca^2+^	Gln 195; Arg 197	0.07	NA			
		B-octylglucoside	Cys 207; Asn 208; Gly 211	0.07				
		Aldehydo-*N*-acetyl-D-glucosamine	Ser 216; Gly 217; Arg 218; Leu 219; Glu 222	0.07				
		3ʹ,5ʹ-cyclic AMP	Ile 93; Asn 97	0.07				
	MeGATA32	Zn^2+^	Asp 266; Gln 270	0.15	Non-specific serine/threonine protein kinase	EC 2.7.11.1	Gly 292	0.14
		Flavin mononucleotide	Cys 266; Trp 271	0.09				
		AMP-pnp	Ala 301; Ala 302; Val 307; Leu 308; Thr 309; Lys 310	0.06				
Group II	MeGATA2	Chlorophyll A	Leu 153; Cys 154; Gly 158; Il 159; Lys 162; Thr 247	0.14	Carbamoyl-phosphate synthase	EC 6.3.5.5	Thr 169	0.064
		Di-mu-sulfido-diiron	Leu 142; Arg 144; Asn 145; Gly 149; Pro 150; Lys 151; Ser 152; Gly 158	0.13				
	MeGATA3	Zn^2+^	Cys 31; Cys 34; Cys 53; Cys 56	0.49	NA			
	MeGATA5	RP-adenosine-3ʹ,5ʹ-cycle-monophosphorothioate	Ser 23; Gln 24; Ser 27	0.06	NA			
		Beta-D-glucose	Arg 132; Ala 134; Cys 158	0.06				
		2ʹ-deoxycytidine 5ʹ-triphosphate	Asp 72; Lys 75	0.06				
	MeGATA7	Thiosulfate(2−)	Asn 194; Glu 135; IIe 198	0.11	Dextransucrase	EC 2.4.1.5	Asn 271	0.077
		Isopropyl-1-beta-D-thiogalactoside	Asn 194; Arg 183; Ser 184; Leu 192; Cys 193	0.11	Triacylglycerol lipase	EC 3.1.1.3	Leu 192	0.077
	MeGATA8	Zn^2+^	Cys 167; Cys 170; Cys 189; Cys 192	0.16	NA			
	MeGATA11	Zn^2+^	Cys 30; Cys 33; Cys 52; Cys 55	0.16	NA			
		*N*-acetylneuraminic acid	Thr 35; Lys 37; Thr 38; Asn 53; Ala 54; Cys 55	0.08				
		Dinuclear copper ion	Cys 30; Ser 50; Cys 52; Gly 56; Ser 59	0.08				
		Methyl 2-acetamido-1,2-dideoxy-1-seleno-beta-D-glucopyranoside	Lys 28; Thr 31; Gly 43; Gly 44	0.08				
	MeGATA21	Nucleic acids	Met 1; Trp 2; Arg 3; Ser 44; Arg 47	0.17	Non-specific serine/threonine protein kinase	EC 2.7.11.1	Leu 12	0.088
		GTP	Il 18; Arg 19	0.12	Glutamyl-tRNA reductase	EC 1.2.1.70	Glue 98	0.08
	MeGATA24	Zn^2+^	Cys 25; Cys 28; Cys 47; Cys 50	0.56	4-alpha-glucanotransferase	EC 2.4.1.25	Gly 51	0.094
	MeGATA29	Zn^2+^	Cys 29; Cys 32; Cys 51; Cys 54	0.23	Protein geranylgeranyltransferase type I	EC 2.5.1.59	Lys 48	0.075
		L-threitol	Cys 54; Arg 57; Lys 61	0.11				
		Nucleic acids	Lys 36; Ala 53	0.07				
	MeGATA31	Zn^2+^	Asn 100; Cys 103; Cys 122; Cys 125	0.35	Citrate (Si)-synthase	EC 2.3.3.1	Thr 106; Gly114; Leu 116	0.074
							Lys 113; Thr 120	0.074
							Gly 117; Asn 123	0.073
	MeGATA33	Zn^2+^	Cys 197; Cys 200	0.26	NA			
	MeGATA34	Beta-D-mannose	Arg 199; Lys 200	0.15	Ribonucleoside-diphosphate reductase	EC 1.17.4.1	Cys 194	0.072
		2ʹ Deoxy-5ʹ-uridylic acid	Cys 194; Gly 195	0.09				
Group III								
	MeGATA15	Zn^2+^	Cys 207; Cys 210; Cys 231; Cys 234	0.15	mRNA guanylyltransferase	EC 2.7.7.50	Ala 189	0.134
					Non-specific serine/threonine protein kinase	EC 2.7.11.1	Leu 230	0.129
	MeGATA16	Cholesterol hydrogen succinate	Ala 232; Leu 235; Phe 236	0.15	Triacylglycerol lipase	EC 3.1.1.3	Ser 215	0.065
		Mg^2+^	Arg 244; Asp 245	0.10	Dextransucrase	EC 2.4.1.5	Asn 231; Gly 234	0.065
	MeGATA17	Cu^2+^	Cys 233; Asn 234; Ala 235; Cys 236	0.19	NA			
	MeGATA18	Ca^2+^	Gly 233; Arg 238	0.06	Oxoglutarate dehydrogenase	EC 1.2.4.2	Glu 28	0.084
		Ca^2+^	Leu 265; Ser 262	0.06	Glutamate--tRNA ligase/Glutamyl-tRNA synthetase H^+^-transporting two-sector ATPase	EC 6.1.1.17	Leu 240	0.081
						EC 3.6.3.14/ EC 7.1.2.2	Ser 226	0.079
	MeGATA26	Xenon	Ser 223; Thr 224; Gly 233; Pro 234; Cys 238; Asn 239	0.08	Glycogen phosphorylase	EC 2.4.1.1	Gly 233	0.081
	MeGATA35	Plastoquinone 9	Val 96; Val 99; Leu 100; Val 120; Leu 103	0.14	Alpha-glucosidase	EC 3.2.1.20	Asp 89; Gln 97	0.09
		Oxalate^2−^	Ser 210	0.09				
	MeGATA36	Plastoquinone 9	Leu 100; Leu 99; Val 93; Val 96	0.16	NA			
		Arginine	Pro 224; Gly 223; Arg 222; Leu 236	0.10				
Group IV	MeGATA1	Zn^2+^	Cys 7; Cys 10; Cys 29; Cys 32	0.18	NA			
	MeGATA4	Zn^2+^	Cys 7; Cys 10; Cys 29; Cys 32	0.17	NA			
		Formamide	Ala 31; Ser 34; Arg 35;	0.20				
	MeGATA19	Cu^1+^	Cys 29; Cys 32	0.27	NA			
		Mg^2+^	Asn 30; Ser 34; Arg 39	0.12				

*The ligands can be searched as Yang *et al.* (2013) or in BioLip database (https://zhanggroup.org/BioLiP/qsearch_pdb.cgi?pdbid=1eth). The information on ligands with high C-score values and on enzyme activity with both high C-score^EC^ values and active amino acid residue sites were shown, where those with C-score or C-score^EC^ values but without definite active amino acid sites were considered to have neither ligand binding nor enzyme activity. C-score, confidence score (0–1), where a higher score indicates a more reliable prediction; EC, enzyme commission; NA, no amino acid residues or no enzyme activities were found.

**Figure 1.**
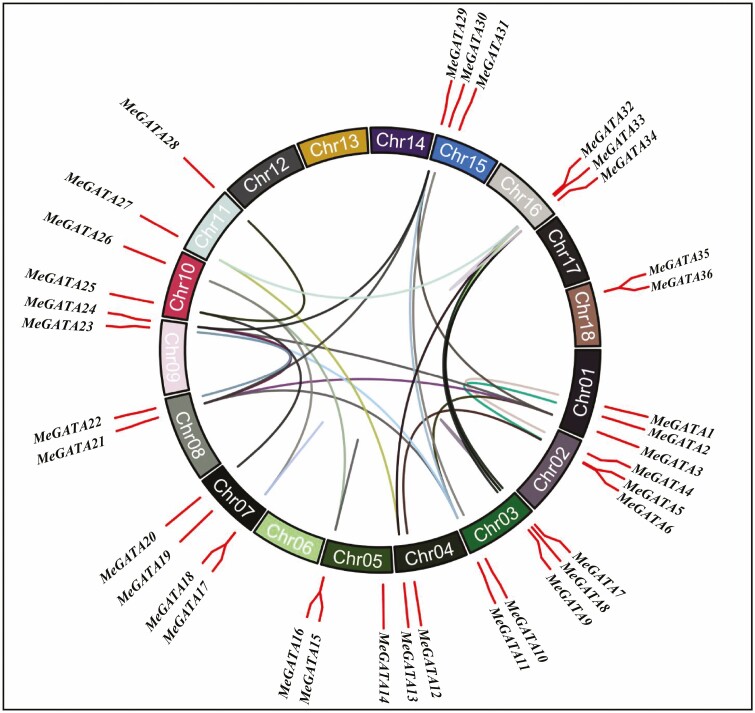
Chromosomal distribution and genome duplication of cassava *MeGATA*s.

*MeGATA* genes were distributed on chromosomes except chromosomes 6, 12–14 and 17 (Fig. 1). The transcript number was up to 4 in *MeGATA10* (Table 1). Seven categories of 97 *cis*-acting elements were found to be present in promoters of *MeGATA*s, which were related to development, environment, hormone, light, promoter, site binding and other elements **[see****Supporting Information—Table S6****]**.

### Evolution of MeGATAs

**Figure 2.**
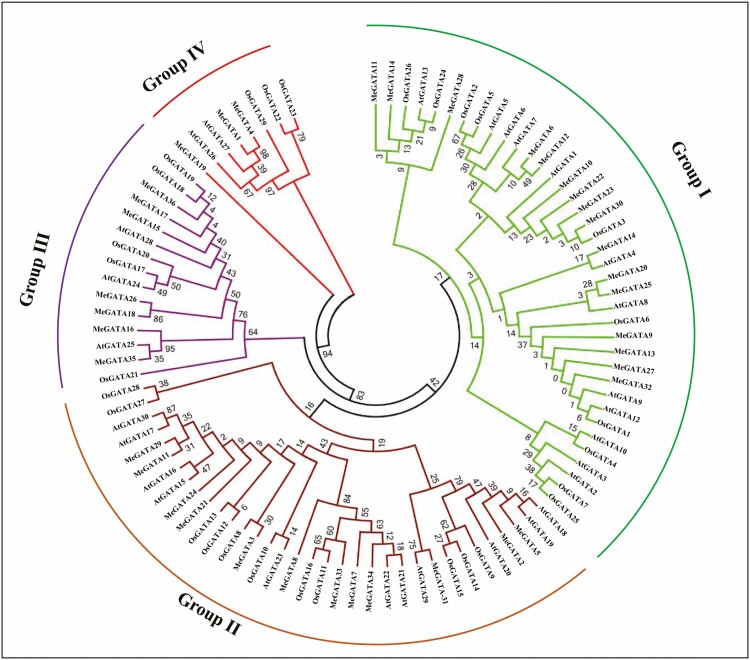
Phylogenetic tree in the primary structures of *MeGATA*s.

A total of 30 *Arabidopsis* AtGATAs **[see****Supporting Information—Table S7****]**, 29 rice OsGATAs **[see****Supporting Information—Table S7****]** and 36 cassava MeGATAs (Table 1) were analysed together. The neighbour-joining tree showed that apart from MeGATA19, which was on an isolated evolutionary branch, the other MeGATAs could be categorized into four groups. Group I was largest and group IV was smallest in member number (Fig. 2).

### Duplication of *MeGATA*s

Duplications of 31 pairs of *MeGATA*s were found, including one pair of tandem-duplicated *MeGATA17–MeGATA18* and 30 pairs of whole genome-duplicated *MeGATA*s (Fig. 1; **see****Supporting Information—Table S8**). The *K*_a_/*K*_s_ values were less than 1, and divergence time was therefore estimated to occur between 9.36 and 112.39 Mya ago **[see****Supporting Information—Table S8****]**.

### Collinearity of *MeGATA*s with GATA motif-containing genes of other plants

**Figure 3.**
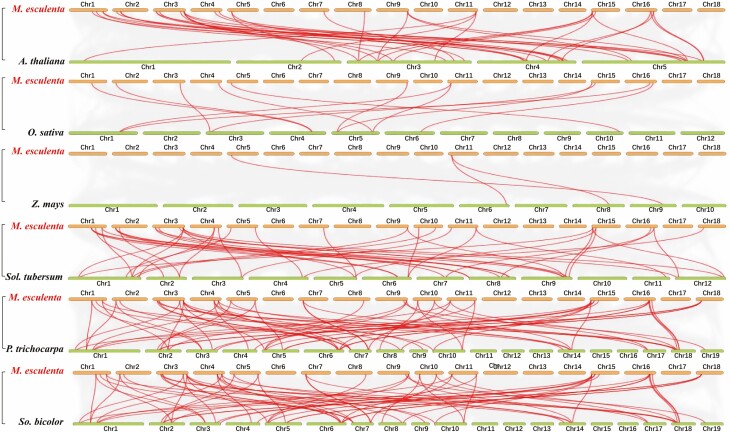
The collinear relationship of *MeGATA*s with orthologous genes of *Arabidopsis*, poplar, and potato, rice, maize, and sorghum. The genes were listed in **Supporting Information—Table S9**. *A.*, *Arabidopsis*; *M*., *Manihot*; *O.*, *Oryza; P*., *Populus*; *So*., *Sorghum*; *Sol*., *Solanum*; *Z.*, *Zea*.

There were 22, 30, and 24 *MeGATA*s which showed a collinearity with orthologous genes of three dicots of *Arabidopsis*, poplar, and potato at the genome level (Fig. 3; **see****Supporting Information—Table S9**), respectively. A total of 11, 2, and 11 *MeGATA*s presented a collinearity with orthologous genes of three monocots of rice, maize and sorghum (Fig. 3; **see****Supporting Information—Table S9**), respectively. *MeGATA14* showed a collinearity with orthologous genes of six monocots and dicots, and six *MeGATA*s (*MeGATA*s *2*, *3*, *5*, *9*, *12* and *18*) had a collinearity only with orthologous genes of monocots (Fig. 3; **see****Supporting Information—Table S9**). The detailed positions of paired GATA motif-containing genes on chromosomes were shown in **Supporting Information—Table S9**.

### MeGATA interactions, and MeGATA-targeted genes

**Figure 4.**
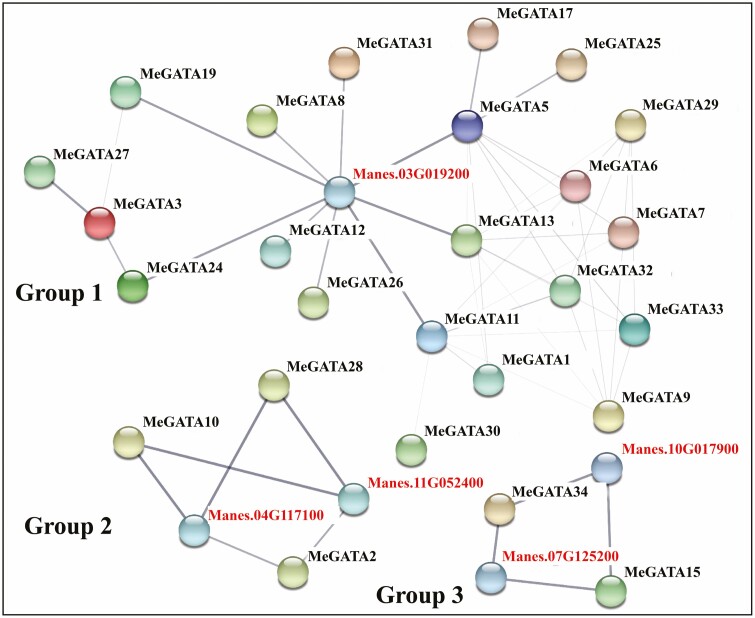
Interaction networks between MeGATAs and other cassava proteins.

Three groups of MeGATA interactions were found, with group 1 of 21 MeGATAs being the largest. In group 1, cassava Manes.03G019200 was in the key point in the network (Fig. 4). In group 2, three MeGATAs (MeGATAs 2, 10 and 28) interacted with two cassava proteins Manes.04G117100 and Manes.11G052400. Group 3 was smallest, involving MeGATA34, MeGATA15, Manes.10G017900 and Manes.07G125200 (Fig. 4).

Although all 36 MeGATAs were analysed, only six MeGATAs (MeGATAs 10, 16, 18, 28, 29 and 32) were found to target and potentially regulate the expression of the other 292 cassava genes **[see****Supporting Information—Table S10****]**, which did not mean that other MeGATAs were not TFs and could not target other downstream genes. This was likely due to the limited organ information allowed to be input in analysis by using the PlantRegMap tool, and the fact that we only chose the FunTFBS method. The GO enrichment-based analysis indicated that all these MeGATA-targeted genes were directed to functional categories of radial pattern formation (GO:0009956) and phyllome development (GO:0048827). The KEGG enrichment-based analysis indicated that they were directed to the path of autophagy (KO04136).

### The miRNA-affected *MeGATA*s and subcellular localization of MeGATAs

**Figure 5.**
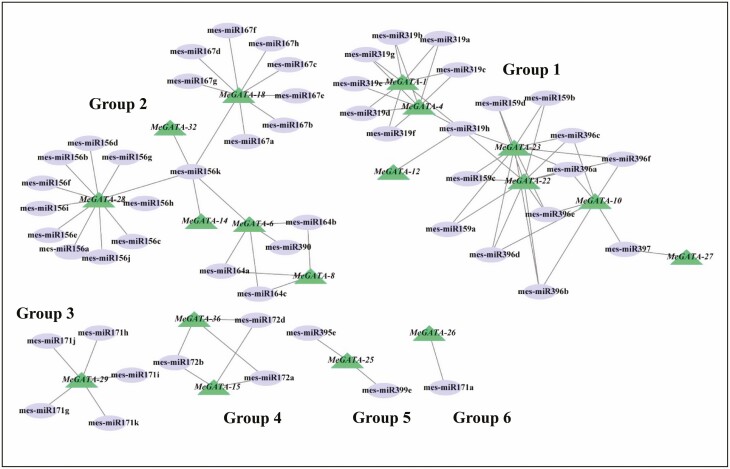
Interaction networks between miRNAs and miRNAs-acted *MeGATA*s. Information on miRNAs and miRNAs-acted *MeGATA*s was shown in **Supporting Information—Table S11**.

A total of 53 putative miRNAs were identified to have the potential for targeting and regulating 18 *MeGATA*s (Fig. 5; **see****Supporting Information—Table S11**). The miRNAs–*MeGATA*s could be grouped into five networks (Fig. 5): group 1 involving seven *MeGATA*s (*MeGATA*s *4*, *7*, *10*, *12*, *22*, *23* and *27*), group 2 involving six *MeGATA*s (*MeGATA*s *6*, *8*, *14*, *18*, *28* and *32*), group 4 involving *MeGATA15* and *MeGATA36*, group 3 involving *MeGATA29*, group 5 involving *MeGATA25,* and group 6 involving *MeGATA26*.

**Figure 6.**
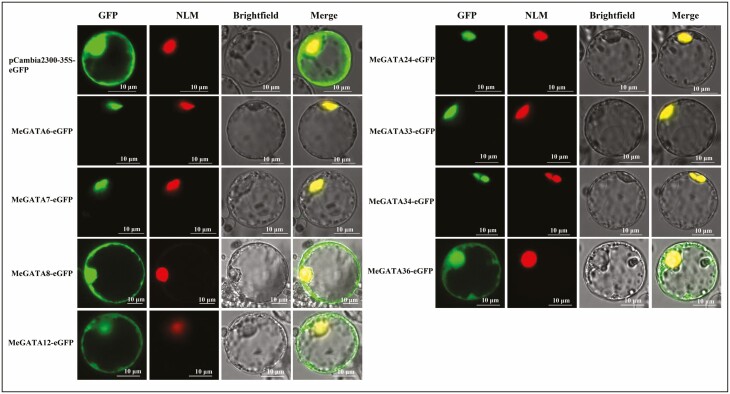
Subcellular localization of MeGATAs. NLM, nuclear localization marker.

The GFP–MeGATAs fusion expression indicated that eight MeGATAs (MeGATAs 6–8, 12, 24, 33, 34 and 36) that were randomly selected were localized in the nucleus (Fig. 6), being consistent with the predicted subcellular localization (Table 1).

### Expression of *MeGATA*s under normal growth conditions

As a whole, 14 *MeGATA*s (*MeGATA*s *2*, *3*, 5, *6*, *8*, *9*, *13*, *19*, *21*, *24*, *27* and *31*–*33*) were expressed at low levels or not expressed, not only in roots, stems and leaves of Arg7 and W14 **[see****Supporting Information—Fig. S1A****]** but also in roots of both Arg7 and KU50 at different developmental stages **[see****Supporting Information—Fig. S1B****]**.

### Expression of *MeGATA*s in pot-grown cassava SC124 under stresses and exogenous hormones

The expression of nine *MeGATA*s (*MeGATA*s *3*, *6*, *7*, *8*, *12*, *24*, *33*, *34* and *36*) that were randomly selected was analysed under treatments involving stress and exogenous hormones. The results showed that their expression was responsive to drought **[see****Supporting Information—Fig. S2****]**, salt **[see****Supporting Information—Fig. S3****]**, high temperature **[see****Supporting Information—Fig. S4****]**, ABA **[see****Supporting Information—Fig. S5****]**, IAA **[see****Supporting Information—Fig. S6****]** and SA **[see****Supporting Information—Fig. S7****]**, roughly echoing the existence of the *cis*-acting elements in the promoter **[see****Supporting Information—Table S6****]**. At first glance, the expression peaked in the middle and late stage of treatments although early responses were found in the few *MeGATA*s **[see****Supporting Information—Figs S1****–****S7****]**.

## Discussion

In this study, 36 GATA genes (*MeGATA*s) were found in cassava, which could be divided into four evolutionary groups (Fig. 2A). The *MeGATA* differed in number from other plant species such as 30 *AtGATA*s in *Arabidopsis* (Reyes *et al.* 2004; Bi *et al.* 2005), 29 *OsGATA*s in rice (Reyes *et al.* 2004), 86 *Bd GATA*s in *Brachypodium distachyon* (Peng *et al.* 2021), 39 *PtrGATA*s in poplar (An *et al.* 2020), 35 *MdGATA*s in apple (Chen *et al.* 2017), 96 *BnGATA*s in *Brassica napus* (Zhu *et al.* 2020), 64 *GmGATA*s in soybean (Zhang *et al.* 2015), and 38 *ZmGATA*s in maize, and 50 *StGATA*s in *Solanum tuberosum* (Jin *et al.* 2017; http://planttfdb.gao-lab.org/). These results strongly suggest again that GATA gene-based mechanisms of growth, development and stress tolerance likely vary with plant species (Scazzocchio 2000; Patient and McGhee 2002; He *et al.* 2007; Behringer and Schwechheimer 2015). The differences in potential ligand binding and enzyme activity (Table 2) in part suggest that the functions of *MeGATA*s have evolutionarily diverged.

Interestingly, as for *cis*-acting elements in promoters and the expression of the *MeGATA*s assayed under treatments, with the exception of *MeGATA6* that was always expressed at relatively low levels, the others were highly expressed under drought **[see****Supporting Information—Fig. S2****]**, of which *MeGATA6*, *MeGATA633* and *MeGATA34* had a drought-responsive element MYB binding site (MBS) **[see****Supporting Information—Table S6****]**. No definite salt-responsive elements were found in the *MeGATA*s **[see****Supporting Information—Table S6****]**, and three *MeGATA*s (*MeGATA6*, *MeGATA7* and *MeGATA34*) were always expressed at low levels, and another six *MeGATA*s were highly expressed under salt **[see****Supporting Information—Fig. S3****]**. Three *MeGATA*, (*MeGATA3, MeGATA12* and *MeGATA24*), were highly expressed, and the other six genes were expressed at relatively low levels under high temperature **[see****Supporting Information—Fig. S4****]**; however, only *MeGATA3* had a low temperature responsiveness (LTR) element **[see****Supporting Information—Table S6****]**. *MeGATA6* had three elements responding to hormones of ABA, MeJA and ethylene **[see****Supporting Information—Table S6****]**, but, its expression was always low under treatment with ABA **[see****Supporting Information—Fig. S5****]**, IAA **[see****Supporting Information—Fig. S6****],** and SA **[see****Supporting Information—Fig. S7****]**. Most of the *MeGATA*s that were expressed at low levels or not expressed in tissues and organs under normal conditions **[see****Supporting Information—Fig. S1****]** had the characteristics of high expression under stress (Figs 2–4) and hormone treatments (Figs 5–7). However, it should be pointed out that *MeGATA6* also had a circadian and root-specific motif I element related to development **[see****Supporting Information—Table S6****]**, but this gene was expressed in stems and leaves and not in roots under normal conditions **[see****Supporting Information—Fig. S1****]**, suggesting that its expression is more strictly condition-controlled, seeming to be a lazy gene expressed in cassava under abiotic stresses and exogenous hormone treatments. Collectively, the results show that elements in the promoters are not the only factor determining *MeGATA* expression.

It has been demonstrated that non-coding RNAs (ncRNAs) including miRNAs and long ncRNAs are important transcriptional regulators (Panni *et al.* 2020). The ncRNAs not only affect RNA biology (such as alternative splicing and diversity in the transcript number) and the development of organisms and but are also related to the occurrence of diseases (Romero-Barrios *et al.* 2018; Dangelmaier and Lal 2020; Panni *et al.* 2020). Usually, miRNAs post-transcriptionally repress the expression of their target mRNAs (Dangelmaier and Lal 2020). Long ncRNAs can activate, repress or otherwise modulate the expression of target genes by epigenetic modifications (Kopp *et al.* 2018; Dangelmaier and Lal 2020; Gil and Ulitsky 2020). Long ncRNAs and miRNAs can also regulate each other through the ‘sponge’ effect (Panni *et al.* 2020). Therefore, 18 *MeGATA*s (Fig. 5) are likely subjected to post-transcriptional regulation and are repressed by miRNAs. It is necessary to point out that the expression of *MeGATA*s in the miRNA–*MeGATA* networks of groups 1, 2 and 4 (Fig. 5) might have miRNA-mediated mutual regulation. Four *MeGATA*s (*MeGATA*s *6*, *8*, *27* and *32*) with low expression or without expression **[see****Supporting Information—Fig. S1****]** happened to be miRNA targets (Fig. 5), suggesting that their expression is strictly controlled by these miRNAs. It should be noted that although *MeGATA12* in group 1 was predicted to be targeted by mes-miR319h (Fig. 5), the high expression in cassava tissues especially in roots **[see****Supporting Information—Fig. S1****]** hints that the expression of mes-miR319h may be repressed by other genes in group 1 (Fig. 5). Anyway, the results strongly indicate that miRNAs may play an important role in driving functional diversification of *MeGATA*s in evolution.

Duplicate genes may lose their functions (non-functionalization), or acquire new functions (new functionalization) or maintain ancestral functions (subfunctionalization) during evolution (Lynch and Conery 2000). Like other GATA genes (Zhang *et al.* 2015; Chen *et al.* 2017; Zhu *et al.* 2020), some *MeGATA*s also showed either whole genome duplication or tandem duplication **[see****Supporting Information—Table S8****]**. These results indicate that duplications are likely one of the driving forces for the formation of new gene members or novel functions of the GATA gene family depending on plant species. The collinearity of some *MeGATA*s with GATA genes in other plants (Fig. 3; **see****Supporting Information—Table S9**) implies that these orthologous genes probably formed after the divergence of dicots and monocots. In addition, duplication of *MeGATA*s (Fig. 1; **see****Supporting Information—Table S8**) was interconnected with their tissue-specific expression patterns **[see****Supporting Information—Fig. S1****]**. Such duplication could make the genes obtain new or tissue-specific expression and functions (Hsueh and Feng 2020).

Polypeptide ligands and their cognate receptors have co-evolved (Hsueh and Feng 2020). The proteins realize their biological functions through directly interacting with other molecules such as ligands with high specificity and affinity (Du *et al.* 2016). Some MeGATAs probably need ligands and some do not (Table 2), implying that the molecular functions and fine regulatory mechanisms of MeGATAs have evolved along diverging pathways.

TFs can participate in metabolic activities either by indirectly regulating the activities of enzymes such as MYBs (Xiao *et al.* 2021) or as enzymes themselves, such as glyceraldehyde-3-phosphate dehydrogenase (Nicholls *et al.* 2012). Starch serves as a determinant of plant fitness because it can be degraded for the biosynthesis of compatible solutes under stress (Thalmann and Santelia 2017) or through induction of stress responsive hormones such as ABA under stress (Thalmann and Santelia 2017). Stress tolerance partially depends on co-ordinated actions among hormones (Verma *et al.* 2016; Jang *et al.* 2020). Both glucanotransferase and dextransucrase, belonging to the GH70 family, are associated with starch degradation, but the latter is an enzyme functionally similar to starch-acting α-amylases that was regarded as an evolutionary intermediate between GH70 family GS enzymes and GH13 family α-amylases (Chen *et al.* 2021). The evidence also showed that a-1,4 glucanotransferases is implicated in amylopectin synthesis (Colleoni *et al.* 1999). A chloroplastic form of α-glucosidase with a neutral pH optimum is thought to function in starch degradation (Monroe *et al.* 1999). Both MeGATA7 and MeGATA16 were probably potential dextransucrases, MeGATA24 was probably a glucanotransferase, and MeGATA35 was maybe an α-glucosidase (Table 2). Two corresponding genes, *MeGATA7* and *MeGATA24*, also showed strong responses to abiotic stresses **[see****Supporting Information—Figs S2****–****S4****]** and exogenous hormones **[see****Supporting Information—Figs S5****–****S7****]**. Coincidentally, three corresponding genes, *MeGATA7*, *MeGATA16* and *MeGATA24*, showed very low or no expression in cassava roots in the absence of stress **[see****Supporting Information—Fig. S1****]**. These results together indicate that *MeGATA7*, *MeGATA16* and *MeGATA24* are the linkers in the interactions between starch degradation, stress tolerance and hormones in cassava roots.

DNA helicases are known for their fundamentally important roles in genomic stability (Dhar *et al.* 2020). Triacylglycerol lipase, such as *Arabidopsis* AtSDP1, has emerged as a key enzyme in lipid turnover in higher plants (Kelly and Feussner 2016), with an involvement in pollen germination and pollen tube growth in *Arabidopsis* (Fan *et al.* 2014). Many protein kinases such as SNF1/AMPK (Mao *et al.* 2010) and *Arabidopsis* Salt-Overly-Sensitive 2 are important enzymes in stress signaling in plants (Zhu 2016). Ribonucleoside-diphosphate reductase catalyses an essential step in DNA biosynthesis (Yasara *et al.* 2021). Inhibition of this enzyme by hydroxyurea depresses the formation of nuclear and plastid DNA, and removal of hydroxyurea from the blocked cells leads to a burst of nuclear DNA synthesis (Heinhorst *et al.* 1985). These examples suggest that MeGATAs may play an array of potential roles in genomic stability and nuclear DNA synthesis (by MeGATA12), mRNA stability (by MeGATA15), pollen development-involved lipid metabolisms (by MeGATA7 and MeGATA16) and sensing stress signals (by MeGATA9, MeGATA20, MeGATA21 and MeGATA25) (Table 2). Taken together with evolutionary commonalities (Fig. 2A) and chromosomal collinearity (Fig. 3), it could be surmised that the functional differentiation trends of GATA genes at least from cassava, *Arabidopsis* and rice would be similar. Although the ligands and enzyme activities (Table 2) were predictive and need to be further confirmed, it does mean that *MeGATA*s are a class of functional generalists.

Anyway, tissue-specific expression **[see****Supporting Information—Fig. S1****]**, responses to abiotic stresses **[see****Supporting Information—Figs S2****–****S4****]** and phytohormones **[see****Supporting Information—Figs S5****–****S7****]**, nuclear localizations (Fig. 6), and *cis*-acting elements in the promoter **[see****Supporting Information—Table S6****]** seem to reinforce a conclusion that GATA TFs are tissue-specific master regulators for induced responses (Block and Shapira 2015).

## Conclusion

There are 36 *MeGATA*s in cassava. Some of the encoded MeGATAs also have the potential to directly participate as enzymes in metabolic catalysis. The evolutionary groups I, II and III are most active in evolution because characteristics of enzymes appear in some MeGATAs. *MeGATA7*, *MeGATA16,* and *MeGATA24* are potential linkers in the cross-talk between starch metabolism, stress tolerance and hormones in cassava roots. The functional diversification of MeGATAs is driven in part by the emergence of enzyme activity and the ability to bind ligands. The expression of some *MeGATA*s is subject to post-transcriptional regulation by miRNAs. Tissue-specific expression may be partly due to *MeGATA* duplication. All in all, *MeGATA*s have highly diverged in function.

## Supporting Information

The following additional information is available in the online version of this article—

**Table S1.** The accession number of the public high-throughput RNA-seq read archives databases submitted by Wang *et al* (2014).

**Table S2.** Primers used in cloning of *MeGATA*s.

**Table S3.** Primers used in construction of gene fusion expression vector.

**Table S4.** Primers used in RT-qPCR analysis of *MeGATA* expression.

**Table S5.** The amino acid sequences of conserved motifs of MeGATAs.

**Table S6.** The potential *cis*-acting elements in the promoter region of *MeGATA*s.

**Table S7.** The ID number of GATA genes in *Arabidopsis* and rice.

**Table S8.** The duplication events of *MeGATA*s.

**Table S9.** The GATA genes in collinearity.

**Table S10.** The cassava genes potentially targeted by MeGATAs.

**Table S11.** Information on miRNAs and miRNAs-targeted MeGATAs.

**Figure S1.** Expression profiles of *MeGATA*s in roots, stems and leaves (A) and in roots at different developmental stages (B) of cassava.

**Figure S2.** Expression profiles of *MeGATA*s in the leaves of cassava SC124 under drought.

**Figure S3.** Expression profiles of *MeGATA*s in the leaves of cassava SC124 under salt.

**Figure S4.** Expression profiles of *MeGATA*s in the leaves of cassava SC124 under high temperature of 42 °C.

**Figure S5.** Expression profiles of *MeGATA*s in the leaves of cassava SC124 under exogenous ABA.

**Figure S6.** Expression profiles of *MeGATA*s in the leaves of cassava SC124 under exogenous IAA.

**Figure S7.** Expression profiles of *MeGATA*s in the leaves of cassava SC124 under exogenous SA.

plac057_suppl_Supplementary_Figure_S1

plac057_suppl_Supplementary_Figure_S2

plac057_suppl_Supplementary_Figure_S3

plac057_suppl_Supplementary_Figure_S4

plac057_suppl_Supplementary_Figure_S5

plac057_suppl_Supplementary_Figure_S6

plac057_suppl_Supplementary_Figure_S7

plac057_suppl_Supplementary_Table_S1

plac057_suppl_Supplementary_Table_S2

plac057_suppl_Supplementary_Table_S3

plac057_suppl_Supplementary_Table_S4

plac057_suppl_Supplementary_Table_S5

plac057_suppl_Supplementary_Table_S6

plac057_suppl_Supplementary_Table_S7

plac057_suppl_Supplementary_Table_S8

plac057_suppl_Supplementary_Table_S9

plac057_suppl_Supplementary_Table_S10

plac057_suppl_Supplementary_Table_S11

## Data Availability

All the data are included in figures, tables and the Supporting **Information**.

## Notes

**Phenome, Genome & Environment. Chief Editor:** Colleen Doherty
