# Supplementary figures and images for: MeGATAs, functional generalists in interactions between cassava growth and development, and abiotic stresses
Source: AoB Plants. 2022 Nov 25;15(1):plac057. doi: 10.1093/aobpla/plac057 (PMC9840210; doi:10.1093/aobpla/plac057)

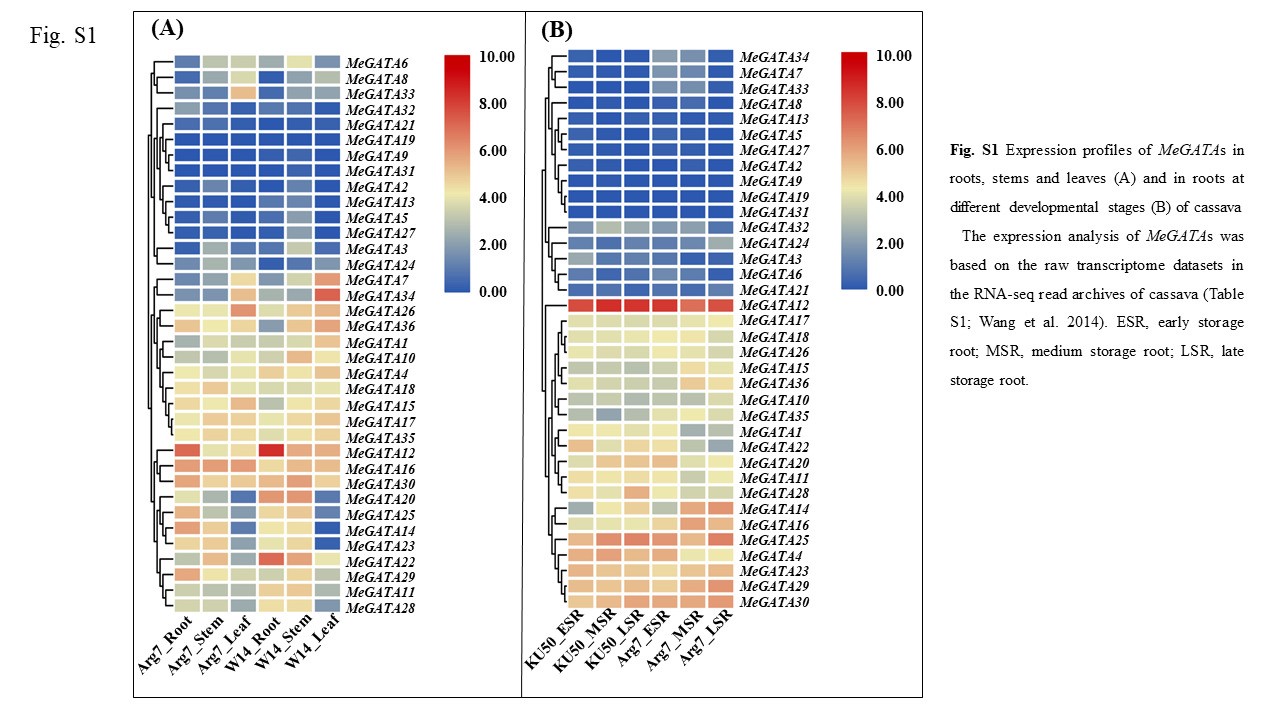

Supplement: plac057_suppl_Supplementary_Figure_S1 [file plac057_suppl_supplementary_figure_s1.jpeg]

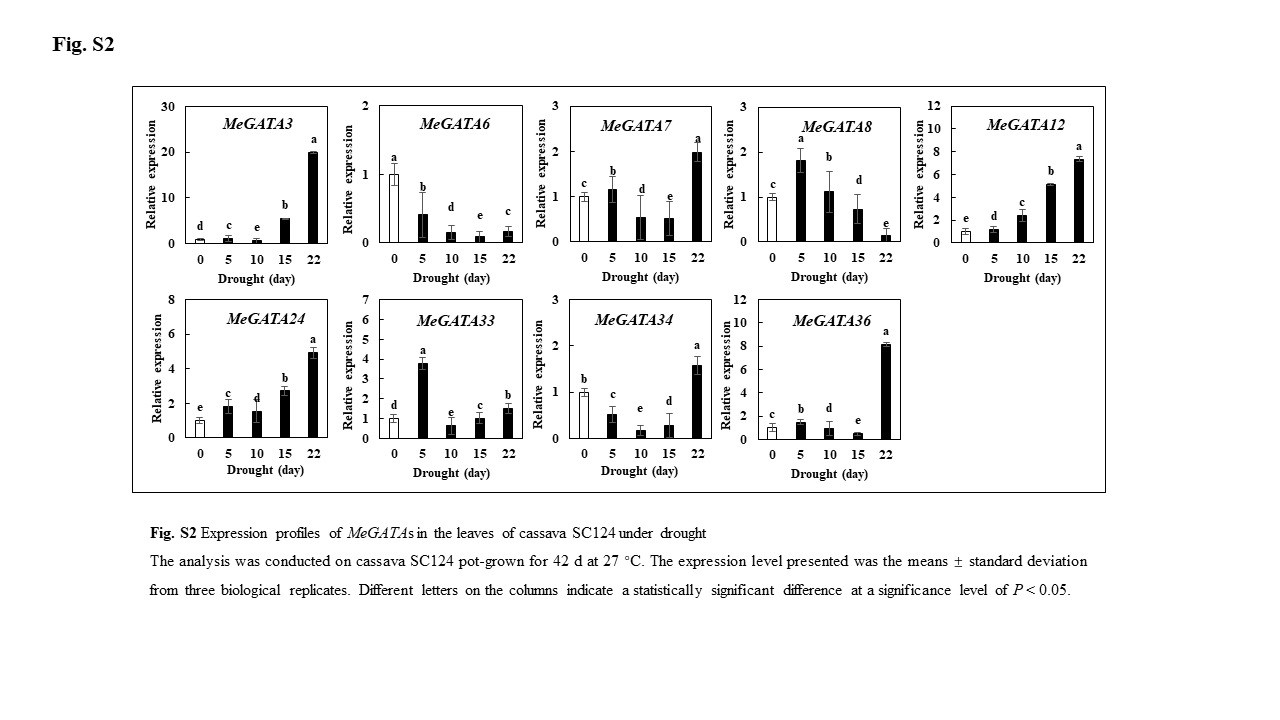

Supplement: plac057_suppl_Supplementary_Figure_S2 [file plac057_suppl_supplementary_figure_s2.jpeg]

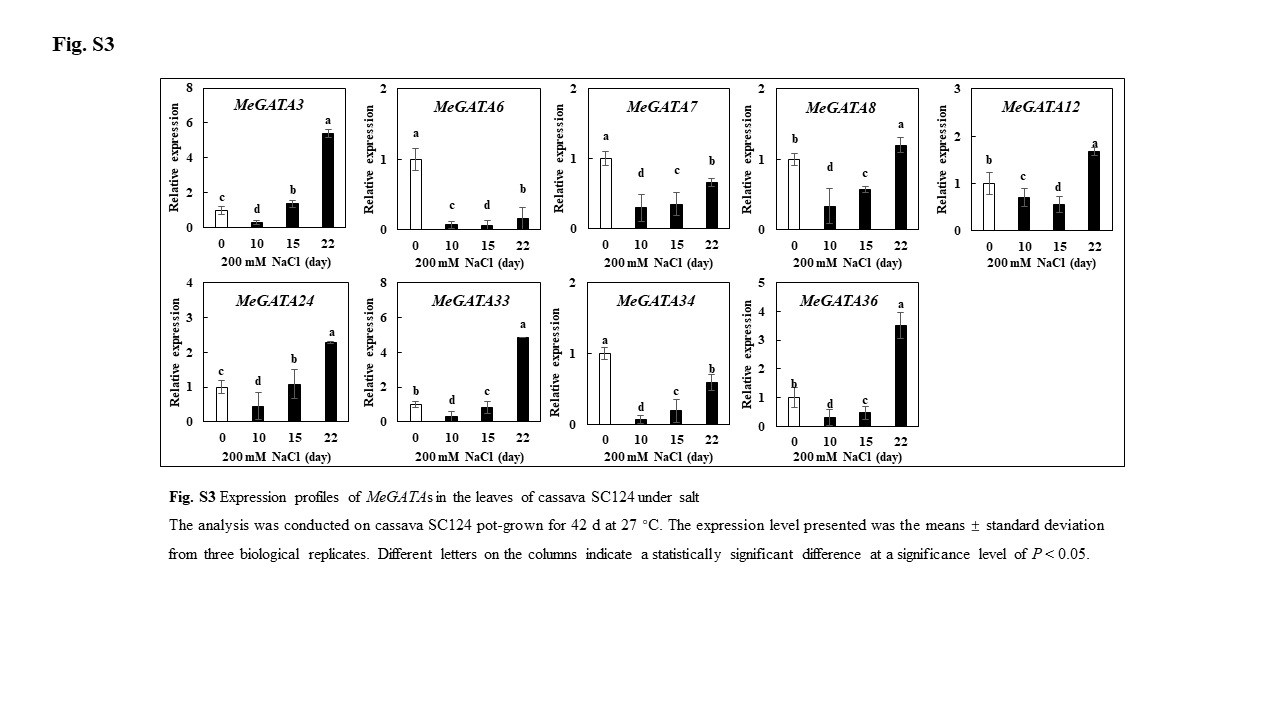

Supplement: plac057_suppl_Supplementary_Figure_S3 [file plac057_suppl_supplementary_figure_s3.jpeg]

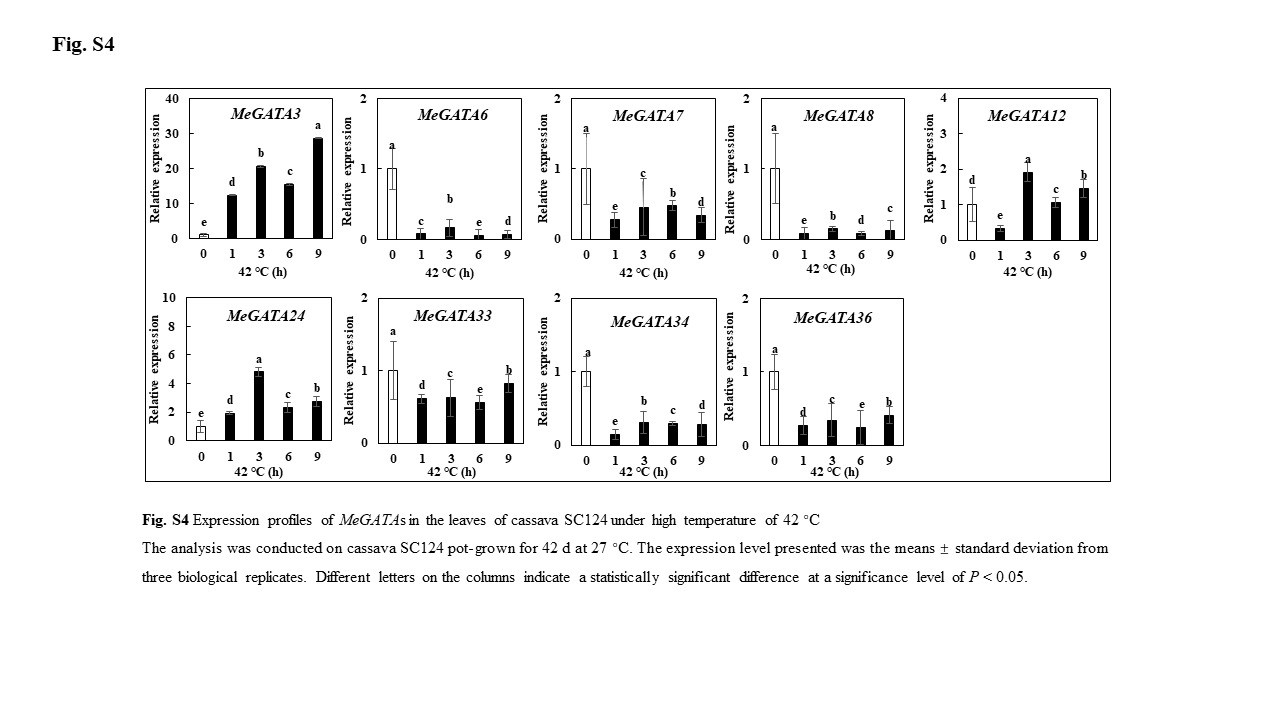

Supplement: plac057_suppl_Supplementary_Figure_S4 [file plac057_suppl_supplementary_figure_s4.jpeg]

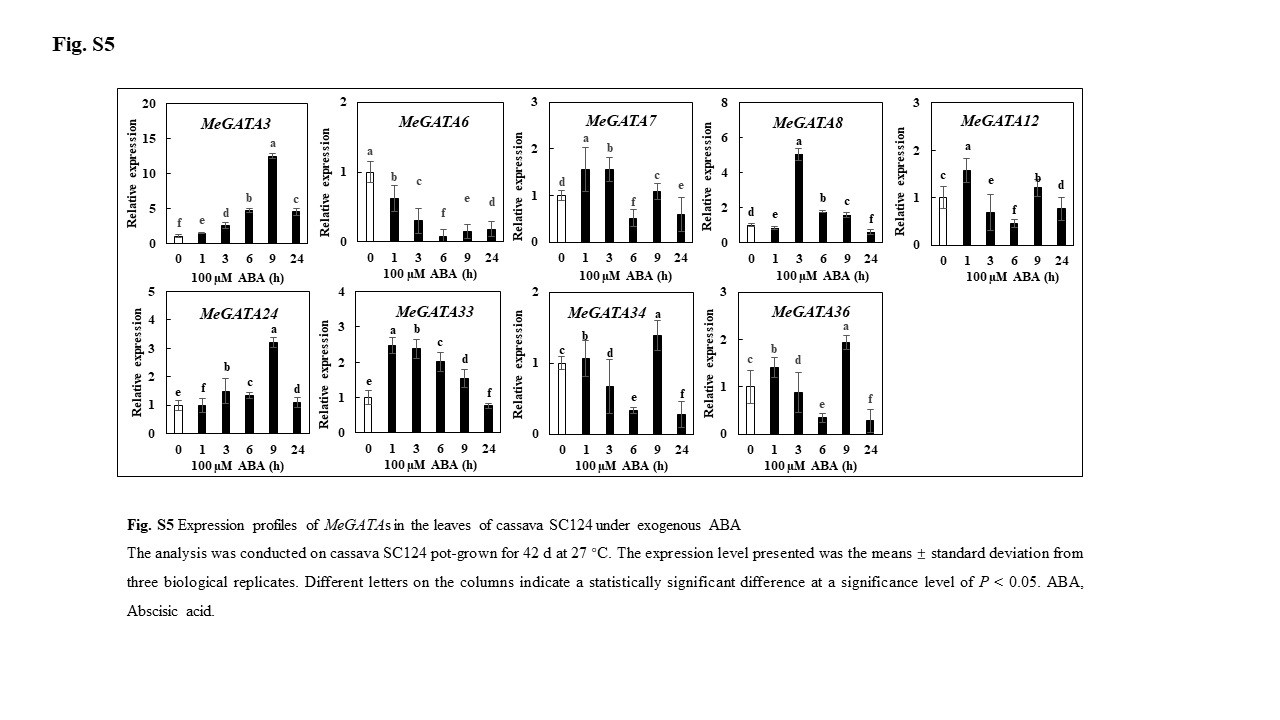

Supplement: plac057_suppl_Supplementary_Figure_S5 [file plac057_suppl_supplementary_figure_s5.jpeg]

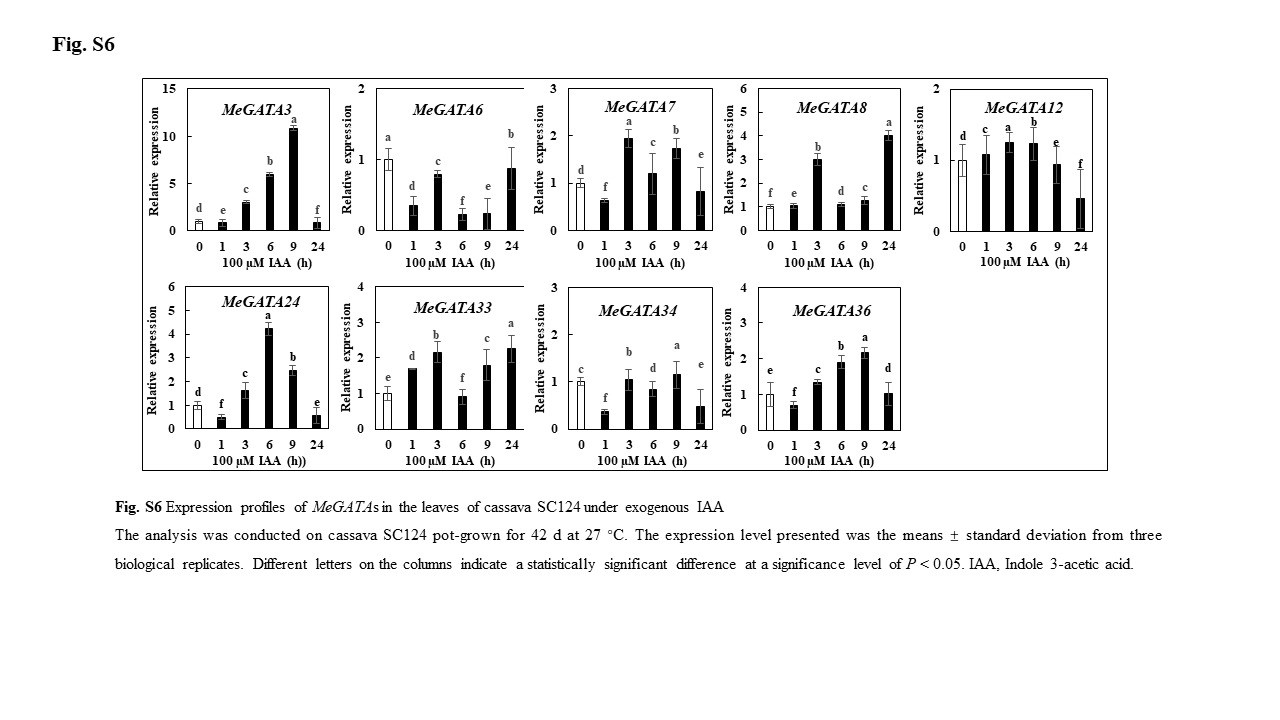

Supplement: plac057_suppl_Supplementary_Figure_S6 [file plac057_suppl_supplementary_figure_s6.jpeg]

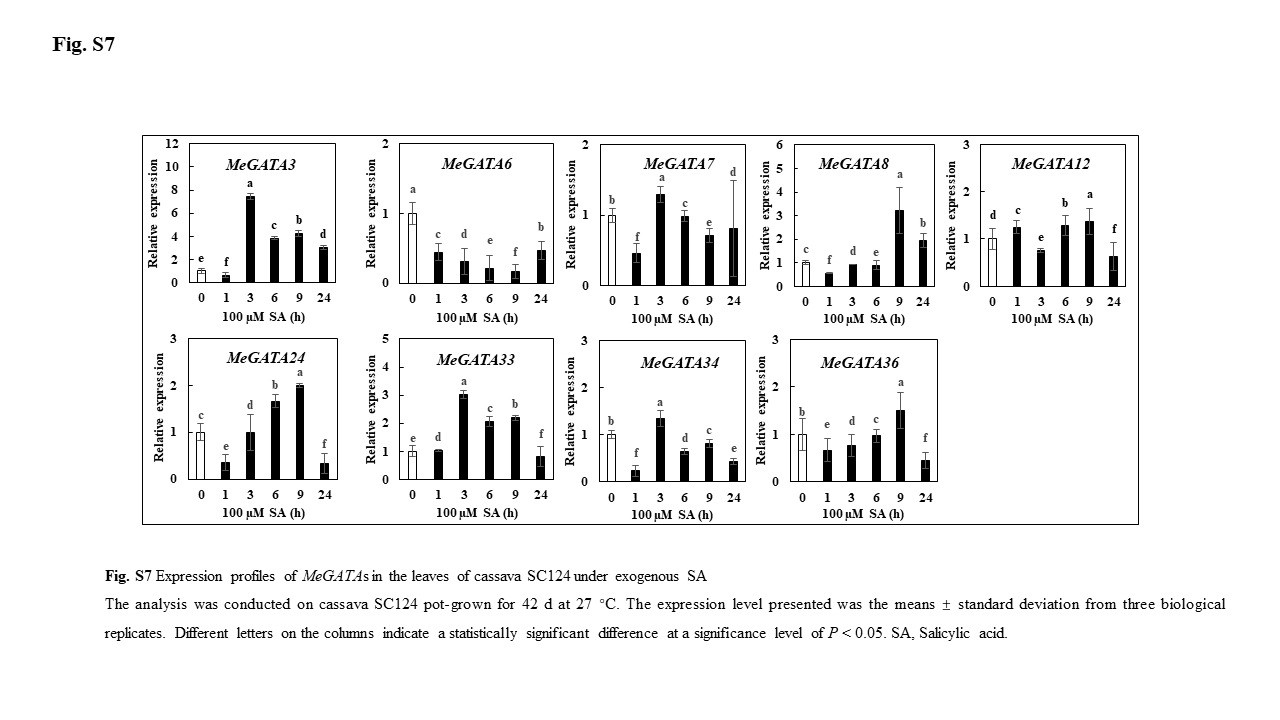

Supplement: plac057_suppl_Supplementary_Figure_S7 [file plac057_suppl_supplementary_figure_s7.jpeg]
