# Supplementary material for: MeGATAs, functional generalists in interactions between cassava growth and development, and abiotic stresses
Source: AoB Plants. 2022 Nov 25;15(1):plac057. doi: 10.1093/aobpla/plac057 (PMC9840210; doi:10.1093/aobpla/plac057)
Supplement: plac057_suppl_Supplementary_Table_S1 [file plac057_suppl_supplementary_table_s1.pdf]

**Table S1** The accession number of the public high-throughput RNA-seq read archives databases submitted by Wang et al (2014)

| **Variety-Tissue** | **Accession number** |
|---|---|
| W14_middle storage root | SRR1298996 |
| W14_leaf | SRR1298998 |
| W14_stem | SRR1298999 |
| Arg7_middle storage root | SRR1299006 |
| Arg7_leaf | SRR1299009 |
| Arg7_stem | SRR1299008 |
| KU50_root ear storage root | SRR1299001 |
| KU50_middle storage root | SRR1299002 |
| KU50_last storage root | SRR1299003 |
| Arg7_early storage root | SRR1299005 |
| Arg7_middle storage root | SRR1299006 |
| Arg7_last storage root | SRR1299007 |

Wang W, Feng B, Xiao J, Xia Z, Zhou X, Li P, Zhang W, Wang Y, Moller BL, Zhang P, Luo MC, Xiao G, Liu J, Yang J, Chen S, Rabinowicz PD, Chen X, Zhang HB, Ceballos H, Lou Q, Zou M, Carvalho LJ, Zeng C, Xia J, Sun S, Fu Y, Wang H, Lu C, Ruan M, Zhou S, Wu Z, Liu H, Kannangara RM, Jorgensen K, Neale RL, Bonde M, Heinz N, Zhu W, Wang S, Zhang Y, Pan K, Wen M, Ma PA, Li Z, Hu M, Liao W, Hu W, Zhang S, Pei J, Guo A, Guo J, Zhang J, Zhang Z, Ye J, Ou W, Ma Y, Liu X, Tallon LJ, Galens K, Ott S, Huang J, Xue J, An F, Yao Q, Lu X, Fregene M, Lopez-Lavalle LA, Wu J, You FM, Chen M, Hu S, Wu G, Zhong S, Ling P, Chen Y, Wang Q, Liu G, Liu B, Li K, Peng M (2014) Cassava genome from a wild ancestor to cultivated varieties. NAT COMMUN 5: 5110
