# Supplementary material for: MeGATAs, functional generalists in interactions between cassava growth and development, and abiotic stresses
Source: AoB Plants. 2022 Nov 25;15(1):plac057. doi: 10.1093/aobpla/plac057 (PMC9840210; doi:10.1093/aobpla/plac057)
Supplement: plac057_suppl_Supplementary_Table_S2 [file plac057_suppl_supplementary_table_s2.pdf]

Table S2 Primers used in cloning of *MeGATA*s

MeGATA	**Forward primer (5′→3′)**	**Reverse primer (5′→3′)**
MeGATA6	ATCGTCTCTCGGTTAATGCTT	TCACGCTGATTTGGCTCTGT
MeGATA7	CATGACTCCTCTTTATCATTCTCCT	TGGTTGCTCACACTCACACA
MeGATA8	CCAGACAATACAGGCTCACCA	TGGTTCGCATCAACTCTCATCA
MeGATA12	AGCCTTCATTTCAGTGCCTCA	CCTGCAAAACATCCCCTTCA
MeGATA24	CTGAATCACAGCCTGGTGGA	AAGAAGAAAGGTAAATGCACCCA
MeGATA33	ATGACTCCATTCTATCTGAATCCAC	AGAAGCTCTCCGAGTCAAGAA
MeGATA34	TTCAGGGCTCTCCTCCTTCA	AGCTCCGCAGATCACAACAC
MeGATA36	GTCGGCCAGGTGCTCTAAG	TGGCATCTCACTCCAAGAACT
