# Supplementary material for: MeGATAs, functional generalists in interactions between cassava growth and development, and abiotic stresses
Source: AoB Plants. 2022 Nov 25;15(1):plac057. doi: 10.1093/aobpla/plac057 (PMC9840210; doi:10.1093/aobpla/plac057)
Supplement: plac057_suppl_Supplementary_Table_S3 [file plac057_suppl_supplementary_table_s3.pdf]

Table S3 Primers used in construction of gene fusion expression vector

MeGATA	**Forward primer (5'→3')**	**Reverse primer (5'→3')**
MeGATA6	CGGGGATCCTCTAGAGTCGACATGCTTT ACCAAACTCACCATCC	CACCATGGTACTAGTGTCGACAAAACTGGG TACAATTGGAGGAGC
MeGATA7	CGGGGATCCTCTAGAGTCGACATGACTC CTCTTTATCATTCTCCTTTT	CACCATGGTACTAGTGTCGACCCCATGAAC AAGGCCATAAGA
MeGATA8	GGACAGGGTACCCGGGGATCCATGACT CCTGTCTGTGATCTGAATCC	AGTGTCGACTCTAGAGGATCCACTATGAAT AAAACCACAAGATAATTCCA
MeGATA12	CGGGGATCCTCTAGAGTCGACATGGAG TATTGCGTGGCTGAG	CACCATGGTACTAGTGTCGACAAAATTAGG AACCATGCCATATTCC
MeGATA24	GGACAGGGTACCCGGGGATCCATGATG GATGATCTCTGGGAAAGG	AGTGTCGACTCTAGAGGATCCTGCAAGAAC TGAGCCACAAGAC
MeGATA33	CGGGGATCCTCTAGAGTCGACATGGAG TCTCTTGACCCAGCAG	CACCATGGTACTAGTGTCGACCCCTTTCTCC ATAGGTTTCACAA
MeGATA34	GGACAGGGTACCCGGGGATCCATGACT CCTGTTTATCATTCTTCTTTTT	AGTGTCGACTCTAGAGGATCCACCATGAAC AAGACCATAAGATAGAGC
MeGATA36	CGGGGATCCTCTAGAGTCGACATGGCG GCGGGAAATCCA	CACCATGGTACTAGTGTCGACTACTACTACT GGATTGGCAGAGGGA
