# Supplementary material for: MeGATAs, functional generalists in interactions between cassava growth and development, and abiotic stresses
Source: AoB Plants. 2022 Nov 25;15(1):plac057. doi: 10.1093/aobpla/plac057 (PMC9840210; doi:10.1093/aobpla/plac057)
Supplement: plac057_suppl_Supplementary_Table_S4 [file plac057_suppl_supplementary_table_s4.pdf]

Table S4 Primers used in RT-qPCR analysis of *MeGATA* expression

Gene	Forward primer (5′→3′)	Reverse primer (5′→3′)	Product length (bp)
MeGATA3	ATTGCCACACCACAAGGACA	GCTCTTCCTTTGTCCAACCC	127
MeGATA6	AATACTCCACACCGTTCCCG	TTCTTGCCCGCTTACTCCTG	141
MeGATA7	GGAATTCGGCAAAGGAAGGC	TCATGGCCGATGCCTTAGAA	100
MeGATA8	CTTTGGAGGAGTGGCCCTTT	TCTCTGTGGAAACAGCCGTG	127
MeGATA12	CCCAATCCCATCCAAGCCAA	TGGAGTTGAAGCAGAGGACG	133
MeGATA24	GCCCCTTCATAGAAAAGGTAGT	GGAGGAGAAGAAGAAAGGTAAATGC	107
MeGATA33	GGGATTCGACAGAGGAAGGC	TGACGTCTCCATGGCAACAA	84
MeGATA34	TGTGGAATCCGGCAAAGGAA	TGGCTGCTATCTCAGGACCA	91
MeGATA36	AGTTCTTGGAGTGAGATGCCA	AGAGGAGCCACCTATCACCT	138
cassava4.1_006776 (internal control gene)	TGGTCAGCACATTTGTTCGT	AGCAGACCCCGTCATTGTAG	106
