# Supplementary material for: MeGATAs, functional generalists in interactions between cassava growth and development, and abiotic stresses
Source: AoB Plants. 2022 Nov 25;15(1):plac057. doi: 10.1093/aobpla/plac057 (PMC9840210; doi:10.1093/aobpla/plac057)
Supplement: plac057_suppl_Supplementary_Table_S5 [file plac057_suppl_supplementary_table_s5.pdf]

Table S5 The amino acid sequences of conserved motifs of MeGATAs

Motif	E-value	Sites	Length (amino acid residue number)	Consensous sequence	Annotation
Motif1	2.8E-696	35	29	[HDG]CGTT[KS]TP[QL][WM]R[RT]GP[ALM]GP[KR][TS]LCNACG[VIL]R[YW][KRA]	Zinc finger, GATA-type (IPR000679)
Motif2	1.40E-295	14	34	SGRL[VFL]PEYRPAASPTF[VS][SLP][TES][LK]HSNSH[RK]KV[LM]E[ML]R[KR][KQ]	-
Motif3	3.70E-186	7	50	[RH][SC][INT][QL][PS][QR]R[AI]ASL[VN]RFR[EQ]KRKERCFDKK[IV]RY[ST]VR[KQ]EVA[LQ]RM[QH]RK[KN]GQF[TA]S[SL]K	CCT domain (IPR010402)
Motif4	1.50E-129	7	46	[VM][GHV][MNR][GA][DGS][RDV][TAS][SD][QE]LTL[ST]F[EQR]G[EQ]VYVF[DP]AV[TS]P[DE]KVQAVLLLLGG[CRY][ED][IL][PT][ST][GA][VP]P	Tify domain (IPR010399)
Motif5	2.00E-103	11	28	EL[CS]VPY[DE]DL[AV][ES]LEWLSNFVEDSFS[SE]ES[LN]	-
Motif6	9.60E-70	8	26	[EAF][HKL]Q[RQ][RV][FK][LP][GQ][DE][EV][EK][EQ]AA[IV]LLM[AE]LS[CY]G[FSL][VI][HSY]	-
Motif7	8.80E-43	13	15	[VI]P[GA][KR]ARSKRSRA[ART][PT][CR]	-
Motif8	1.20E-40	11	15	HFI[VI]DDLLDF[SP]N[DE]DG	-
Motif9	7.80E-35	6	40	[GR][AG][AGP][AEI][DEQ][SI][IE]D[DHN][DHP][GHN][IG][EHR]YE[DN]G[NT][AG][TM][GD][DGV][VG]V[ED]DV[MPS][NP][DS]S[VI][NY]V[APT]S[GH]G[DA][GY]	-
Motif10	2.00E-29	10	29	[LDE][LT][NKR][EA][DEQ]QQ[HQ][LQS][QLV][LKQT][FPY][LI][SEHP][HKLPQ][HGSV][QDET][AENT][AEM][AKV][SFLN][DPL][SV][SD][SIN][GR][SDN][ADM][FY]	-
Motif11	4.80E-23	4	21	W[GNS][GAV][GT]Q[DG]SGQDD[SI][QIM][QLV]ET[SLT]CTHC	-
Motif12	7.30E-22	2	50	MMHRC[CS]SSQGNMMGQCTCGLF[HP]SQ[NS]NSF[ST]MLFSMPNH[HN][KN]SFDEADMYPFA	-
Motif13	2.80E-20	4	21	E[GN]S[AS][AV]KW[MT][PS][SA][KR]MRLM[HKQR][KR]MM[NS]S	-
Motif14	3.70E-19	2	50	DLTGPAQSIVWD[ST]MVPSRKRTCVNR[PT]KPSPVEKLTKDLYTIWHEQQSSCF	-
Motif15	1.30E-16	2	50	DDYEDHRVSRGKSISINKNKDVKLLKRK[AV]NYDNGVV[DG][GR][FI]APDY[NY]QGYRKV	-
Motif16	8.50E-16	6	30	K[AR][RS]R[AD]MA[AG][AQ][AK]A[GP][AE]N[GK][TV][IM][FKV][SG]P[EK]R[SI][AN]MKT[KT][MY][EH]	-
Motif17	8.70E-16	5	14	[LFI][IY]H[HQ]H[SLMV][GH]P[DNK][FQ][RN][HQF][LEIM]I	-
Motif18	2.70E-11	2	50	LFESETPMVSVEIGHGSVLIRHPSSIARDEESEASSLSVENKQYS[IT][NS]EAY	-
Motif19	2.20E-11	2	42	VDCTLSLGTPSTRLSED[DE]DKR[IS]RHERRPSSCMSNFCWDILQT	-
Motif20	3.40E-10	2	50	NKEVMD[NS]DGSCFSPRSLFALPPDGGSLMLD[ST][FL]HYVDESSDQDLLL[DH]VPSN	-
