# Supplementary material for: MeGATAs, functional generalists in interactions between cassava growth and development, and abiotic stresses
Source: AoB Plants. 2022 Nov 25;15(1):plac057. doi: 10.1093/aobpla/plac057 (PMC9840210; doi:10.1093/aobpla/plac057)
Supplement: plac057_suppl_Supplementary_Table_S6 [file plac057_suppl_supplementary_table_s6.pdf]

Table S6 The potential *cis*-acting elements in the promoter region of *MeGATA*s

**Classification**	**Name**	**Functions**	***MeGATA***
Development-related elements	CAT-box	Cis acting regulatory element related to meristem expression	*MeGATA2, MeGATA5, MeGATA11, MeGATA17, MeGATA23, MeGATA24, MeGATA32, MeGATA36*
	CCGTCC box	Cis acting regulatory element related to meristem specific activation	*MeGATA5, MeGATA13, MeGATA16, MeGATA23, MeGATA32*
	Circadian	Cis acting regulatory element involved in circadian control	*MeGATA1, MeGATA3, eGATA6M, MeGATA8, MeGATA24, MeGATA31, MeGATA34*
	GCN4_motif	Cis regulatory element involved in endosperm expression	*MeGATA2, MeGATA24*
	HDZip 1	element involved in differentiation of the palisade mesophyll cells	*MeGATA9, MeGATA22*
	Motif I	Cis acting regulatory element root specific	*MeGATA6*
	MSA like	Cis acting element involved in cell cycle regulation	*MeGATA29*
	O2 site	Cis acting regulatory element involved in zein metabolism regulation	*MeGATA1, MeGATA2, MeGATA7, MeGATA10, MeGATA11, MeGATA12, MeGATA22, MeGATA24*
	RY element	Cis acting regulatory element involved in seed specific regulation	*MeGATA19, MeGATA22*
Environmental stress-related elements	ARE	Cis acting regulatory element essential for the anaerobic induction	*MeGATA1, MeGATA3, MeGATA5, MeGATA7, MeGATA8, MeGATA12, MeGATA14, MeGATA16, MeGATA18, MeGATA20, MeGATA21, MeGATA22, MeGATA23, MeGATA24, MeGATA26, MeGATA29, MeGATA30, MeGATA31, MeGATA34, MeGATA35*
	GC motif	Enhancer like element involved in anoxic specific inducibility	*MeGATA3, MeGATA4, MeGATA26, MeGATA36*
	LTR	Cis acting element involved in low temperature responsiveness	*MeGATA1, MeGATA3, MeGATA7, MeGATA10, MeGATA16, MeGATA18, MeGATA20, MeGATA22, MeGATA26, MeGATA29, MeGATA32, MeGATA36*
	MBS	MYB binding site involved in drought inducibility	*MeGATA2, MeGATA5, MeGATA6, MeGATA10, MeGATA12, MeGATA14, MeGATA18, MeGATA23, MeGATA28, MeGATA33, MeGATA34, MeGATA35*
	TC rich repeats	Cis acting element involved in defense and stress responsiveness	*MeGATA1, MeGATA7, MeGATA8, MeGATA13, MeGATA14, MeGATA18, MeGATA23, MeGATA27, MeGATA29, MeGATA30, MeGATA33*
	WUN motif	Wound responsive element	*MeGATA4, MeGATA5, MeGATA6, MeGATA8, MeGATA9, MeGATA10, MeGATA13, MeGATA14, MeGATA16, MeGATA17, MeGATA18, MeGATA19, MeGATA20, MeGATA22, MeGATA23, MeGATA25, MeGATA30, MeGATA31*
Hormone responsive elements	ABRE	Cis acting element involved in the abscisic acid responsiveness	*MeGATA2, MeGATA3, MeGATA4, MeGATA5, MeGATA6, MeGATA7, MeGATA8, MeGATA10, MeGATA12, MeGATA14, MeGATA18, MeGATA20, MeGATA21, MeGATA22, MeGATA24, MeGATA26, MeGATA27, MeGATA28, MeGATA29, MeGATA30, MeGATA32, MeGATA33, MeGATA34, MeGATA35, MeGATA36*
	AuxRR core	Cis acting regulatory element involved in auxin responsiveness	*MeGATA2, MeGATA5, MeGATA12, MeGATA31*
	CGTCA motif	Cis acting regulatory element involved in the MeJA responsive	*MeGATA6, MeGATA9, MeGATA10, MeGATA11, MeGATA12, MeGATA13, MeGATA18, MeGATA21, MeGATA23, MeGATA24, MeGATA28, MeGATA29, MeGATA30, MeGATA31, MeGATA32, MeGATA34, MeGATA35*
	ERE	Ethylene responsive element	*MeGATA2, MeGATA3, MeGATA4, MeGATA6, MeGATA8, MeGATA9, MeGATA11, MeGATA12, MeGATA14, MeGATA16, MeGATA17, MeGATA18, MeGATA19, MeGATA21, MeGATA22, MeGATA23, MeGATA24, MeGATA25, MeGATA26, MeGATA28, MeGATA29, MeGATA31, MeGATA34*
	GARE motif	Gibberellin responsive element	*MeGATA11, MeGATA24, MeGATA26*
	P box	Gibberellin responsive element	*MeGATA1, MeGATA3, MeGATA4, MeGATA10, MeGATA26, MeGATA28, MeGATA29, MeGATA31, MeGATA33, MeGATA34*
	TATC box	Cis acting element involved in gibberellin responsiveness	*MeGATA32, MeGATA34*
	TCA element	Cis acting element involved in salicylic acid responsiveness	*MeGATA3, MeGATA8, MeGATA12, MeGATA15, MeGATA16, MeGATA22, MeGATA23, MeGATA24, MeGATA27, MeGATA32, MeGATA33*
	TGA box	part of an auxin responsive element	*MeGATA12*
	TGACG motif	Cis acting regulatory element involved in the MeJA responsiveness	*MeGATA6, MeGATA9, MeGATA10, MeGATA11, MeGATA12, MeGATA13, MeGATA18, MeGATA21, MeGATA23, MeGATA24, MeGATA28, MeGATA29, MeGATA30, MeGATA31, MeGATA32, MeGATA34, MeGATA35*
	TGA element	Auxin responsive element	*MeGATA2, MeGATA3, MeGATA5, MeGATA9, MeGATA12, MeGATA16, MeGATA28, MeGATA35*
Light responsive elements	3AF1 binding site	light responsive element	*MeGATA1, MeGATA14, MeGATA28*
	AAAC motif	light responsive element	*MeGATA9*
	ACE	Cis acting element involved in light responsiveness	*MeGATA6, MeGATA21, MeGATA35*
	AE box	part of a module for light response	*MeGATA1, MeGATA4, MeGATA6, MeGATA7, MeGATA8, MeGATA10, MeGATA18, MeGATA20, MeGATA22, MeGATA26, MeGATA34*
	AT1 motif	part of a light responsive module	*MeGATA7, MeGATA11, MeGATA14, MeGATA16, MeGATA17, MeGATA31*
	ATC motif	part of a conserved DNA module involved in light responsiveness	*MeGATA34*
	ATCT motif	part of a conserved DNA module involved in light responsiveness	*MeGATA9, MeGATA11, MeGATA29, MeGATA30*
	Box 4	part of a conserved DNA module involved in light responsiveness	*MeGATA1, MeGATA2, MeGATA3, MeGATA4, MeGATA5, MeGATA6, MeGATA7, MeGATA8, MeGATA9, MeGATA10, MeGATA11, MeGATA13, MeGATA14, MeGATA15, MeGATA16, MeGATA17, MeGATA18, MeGATA19, MeGATA20, MeGATA21, MeGATA22, MeGATA23, MeGATA24, MeGATA25, MeGATA26, MeGATA27, MeGATA29, MeGATA30, MeGATA31, MeGATA32, MeGATA33, MeGATA34, MeGATA35, MeGATA36*
	Box II	part of a light responsive element	*MeGATA21, MeGATA24, MeGATA27*
	CAG motif	part of a light response element	*MeGATA19, MeGATA36*
	chsCMA1a	part of a light responsive element	*MeGATA9, MeGATA12, MeGATA13, MeGATA15, MeGATA19, MeGATA28, MeGATA29, MeGATA31*
	chsCMA2a	part of a light responsive element	*MeGATA4, MeGATA7, MeGATA22, MeGATA29*
	GA motif	part of a light responsive element	*MeGATA5, MeGATA10, MeGATA26, MeGATA34*
	Gap box	part of a light responsive element	*MeGATA15, MeGATA28, MeGATA31*
	GATA motif	part of a light responsive element	*MeGATA2, MeGATA3, MeGATA6, MeGATA7, MeGATA8, MeGATA9, MeGATA11, MeGATA13, MeGATA14, MeGATA23, MeGATA24, MeGATA26, MeGATA32, MeGATA33, MeGATA34, MeGATA36*
	G Box	Cis acting regulatory element involved in light responsiveness	*MeGATA2, MeGATA3, MeGATA4, MeGATA5, MeGATA6, MeGATA7, MeGATA8, MeGATA10, MeGATA12, MeGATA13, MeGATA20, MeGATA21, MeGATA22, MeGATA23, MeGATA24, MeGATA26, MeGATA27, MeGATA28, MeGATA29, MeGATA30, MeGATA32, MeGATA33, MeGATA34, MeGATA35, MeGATA36*
	GT1motif	light responsive element	*MeGATA1, MeGATA2, MeGATA4, MeGATA6, MeGATA7, MeGATA8, MeGATA11, MeGATA13, MeGATA14, MeGATA15, MeGATA17, MeGATA19, MeGATA23, MeGATA24, MeGATA27, MeGATA30, MeGATA32, MeGATA34, MeGATA35*
	GTGGC motif	part of a light responsive element	*MeGATA10, MeGATA25, MeGATA30*
	I box	part of a light responsive element	*MeGATA2, MeGATA3, MeGATA8, MeGATA14, MeGATA15, MeGATA22, MeGATA25, MeGATA27, MeGATA28, MeGATA34, MeGATA36*
	LAMP element	part of a light responsive element	*MeGATA12, MeGATA27, MeGATA28*
	L box	part of a light responsive element	*MeGATA20, MeGATA25*
	MRE	MYB binding site involved in light responsiveness	*MeGATA3, MeGATA9, MeGATA13, MeGATA15, MeGATA22, MeGATA28, MeGATA35*
	Sp1	light responsive element	*MeGATA2, MeGATA10, MeGATA12, MeGATA14, MeGATA16, MeGATA18, MeGATA20*
	TCCC motif	part of a light responsive element	*MeGATA11, MeGATA14, MeGATA17, MeGATA20, MeGATA23, MeGATA31, MeGATA35*
	TCT motif	part of a light responsive element	*MeGATA2, MeGATA3, MeGATA7, MeGATA8, MeGATA9, MeGATA13, MeGATA14, MeGATA16, MeGATA19, MeGATA24, MeGATA26, MeGATA27, MeGATA30, MeGATA32*
Promoter related elements	A box	Cis acting regulatory element	*MeGATA5, MeGATA13, MeGATA16, MeGATA23, MeGATA32*
	AT rich sequence	element for maximal elicitor mediated activation (2copies)	*MeGATA4, MeGATA9, MeGATA20*

	CAAT box	common Cis acting element in promoter and enhancer regions	*MeGATA1, MeGATA2, MeGATA3, MeGATA4, MeGATA5, MeGATA6, MeGATA7, MeGATA8, MeGATA9, MeGATA10, MeGATA11, MeGATA12, MeGATA13, MeGATA14, MeGATA15, MeGATA16, MeGATA17, MeGATA18, MeGATA19, MeGATA20, MeGATA21, MeGATA22, MeGATA23, MeGATA24, MeGATA25, MeGATA26, MeGATA27, MeGATA28, MeGATA29, MeGATA30, MeGATA31, MeGATA32, MeGATA33, MeGATA34, MeGATA35, MeGATA36*
	TATA box	core promoter element around 30 of transcription start	*MeGATA1, MeGATA2, MeGATA3, MeGATA4, MeGATA5, MeGATA6, MeGATA7, MeGATA8, MeGATA9, MeGATA10, MeGATA11, MeGATA12, MeGATA13, MeGATA14, MeGATA15, MeGATA16, MeGATA17, MeGATA18, MeGATA19, MeGATA20, MeGATA21, MeGATA22, MeGATA23, MeGATA24, MeGATA25, MeGATA26, MeGATA27, MeGATA28, MeGATA29, MeGATA30, MeGATA31, MeGATA32, MeGATA33, MeGATA34, MeGATA35, MeGATA36*
Site binding related elements	AT rich element	binding site of AT rich DNA binding protein (ATBP1)	*MeGATA9, MeGATA14, MeGATA16, MeGATA17, MeGATA18, MeGATA27, MeGATA30, MeGATA31*
	Box III	protein binding site	*MeGATA13, MeGATA35*
	CCAAT box	MYBHv1 binding site	*MeGATA4, MeGATA10, MeGATA26, MeGATA27, MeGATA30, MeGATA32, MeGATA36*
	HDZip 3	protein binding site	*MeGATA31, MeGATA32*
	MBSI	MYB binding site involved in flavonoid biosynthetic genes regulation	*MeGATA6, MeGATA15, MeGATA28*
Other elements	AAGAA motif	Unknown	*MeGATA1, MeGATA2, MeGATA3, MeGATA4, MeGATA6, MeGATA8, MeGATA9, MeGATA11, MeGATA12, MeGATA13, MeGATA14, MeGATA18, MeGATA19, MeGATA20, MeGATA21, MeGATA22, MeGATA23, MeGATA24, MeGATA25, MeGATA26, MeGATA27, MeGATA28, MeGATA29, MeGATA30, MeGATA31, MeGATA32, MeGATA34, MeGATA35, MeGATA36*
	ABRE2	Unknown	*MeGATA21, MeGATA24, MeGATA27*
	ABRE3a	Unknown	*MeGATA2, MeGATA3, MeGATA5, MeGATA6, MeGATA20, MeGATA28, MeGATA32, MeGATA35*
	ABRE4	Unknown	*MeGATA2, MeGATA3, MeGATA5, MeGATA6, MeGATA20, MeGATA28, MeGATA32, MeGATA35*
	ACI	Unknown	*MeGATA20, MeGATA25*
	AP1	Unknown	*MeGATA13*
	as1	Unknown	*MeGATA6, MeGATA9, MeGATA10, MeGATA11, MeGATA12, MeGATA13, MeGATA18, MeGATA21, MeGATA23, MeGATA24, MeGATA28, MeGATA29, MeGATA30, MeGATA31, MeGATA32, MeGATA34, MeGATA35*
	box S	Unknown	*MeGATA12, MeGATA24, MeGATA36*
	CARE	Unknown	*MeGATA13*
	CCGTCC motif	Unknown	*MeGATA5, MeGATA13, MeGATA16, MeGATA23, MeGATA32*
	CTAG motif	Unknown	*MeGATA21*
	DRE core	Unknown	*MeGATA2, MeGATA9, MeGATA14, MeGATA18, MeGATA22, MeGATA23, MeGATA36*
	DRE1	Unknown	*MeGATA6, MeGATA9, MeGATA14, MeGATA28, MeGATA36*
	E2Fb	Unknown	*MeGATA12*
	F box	Unknown	*MeGATA24, MeGATA30*
	MYB	Unknown	*MeGATA1, MeGATA2, MeGATA3, MeGATA4, MeGATA5, MeGATA6, MeGATA7, MeGATA8, MeGATA9, MeGATA10, MeGATA11, MeGATA12, MeGATA13, MeGATA14, MeGATA15, MeGATA16, MeGATA18, MeGATA19, MeGATA20, MeGATA21, MeGATA22, MeGATA23, MeGATA24, MeGATA25, MeGATA26, MeGATA27, MeGATA28, MeGATA29, MeGATA30, MeGATA32, MeGATA33, MeGATA34, MeGATA35, MeGATA36*
	MYB recognition site	Unknown	*MeGATA4, MeGATA10, MeGATA26, MeGATA27, MeGATA30, MeGATA32, MeGATA36*
	Myb binding site	Unknown	*MeGATA1, MeGATA11, MeGATA12, MeGATA13, MeGATA24, MeGATA26, MeGATA29, MeGATA34, MeGATA35*
	MYB like sequence	Unknown	*MeGATA1, MeGATA5, MeGATA6, MeGATA7, MeGATA8, MeGATA11, MeGATA12, MeGATA13, MeGATA20, MeGATA21, MeGATA23, MeGATA24, MeGATA25, MeGATA27, MeGATA28, MeGATA30, MeGATA32, MeGATA34, MeGATA36*
	Myc	Unknown	*MeGATA1, MeGATA2, MeGATA3, MeGATA4, MeGATA5, MeGATA6, MeGATA7, MeGATA8, MeGATA9, MeGATA10, MeGATA11, MeGATA12, MeGATA13, MeGATA14, MeGATA15, MeGATA17, MeGATA19, MeGATA20, MeGATA21, MeGATA22, MeGATA23, MeGATA24, MeGATA25, MeGATA26, MeGATA27, MeGATA28, MeGATA30, MeGATA31, MeGATA32, MeGATA33, MeGATA34, MeGATA35, MeGATA36*
	NON	Unknown	*MeGATA10, MeGATA30*
	STRE	Unknown	*MeGATA1, MeGATA2, MeGATA3, MeGATA5, MeGATA7, MeGATA9, MeGATA10, MeGATA11, MeGATA12, MeGATA13, MeGATA14, MeGATA16, MeGATA17, MeGATA18, MeGATA20, MeGATA21, MeGATA23, MeGATA24, MeGATA25, MeGATA26, MeGATA27, MeGATA28, MeGATA29, MeGATA30, MeGATA31, MeGATA32, MeGATA33, MeGATA35, MeGATA36*
	TATA box	Unknown	*MeGATA2, MeGATA3, MeGATA4, MeGATA6, MeGATA7, MeGATA9, MeGATA10, MeGATA11, MeGATA13, MeGATA14, MeGATA15, MeGATA17, MeGATA18, MeGATA21, MeGATA23, MeGATA24, MeGATA26, MeGATA29, MeGATA30, MeGATA32, MeGATA33, MeGATA34, MeGATA36*
	TCA	Unknown	*MeGATA2, MeGATA4, MeGATA5, MeGATA7, MeGATA9, MeGATA10, MeGATA11, MeGATA12, MeGATA14, MeGATA20, MeGATA21, MeGATA22, MeGATA24, MeGATA25, MeGATA27, MeGATA29*
	Telo box	Unknown	*MeGATA16, MeGATA35*
	Unnamed__1	Unknown	*MeGATA1, MeGATA2, MeGATA5, MeGATA6, MeGATA7, MeGATA10, MeGATA11, MeGATA12, MeGATA14, MeGATA15, MeGATA16, MeGATA17, MeGATA18, MeGATA20, MeGATA21, MeGATA24, MeGATA26, MeGATA27, MeGATA28, MeGATA29, MeGATA30, MeGATA31, MeGATA32, MeGATA34, MeGATA35, MeGATA36*
	Unnamed__10	Unknown	*MeGATA12*
	Unnamed__12	Unknown	*MeGATA12*
	Unnamed__14	Unknown	*MeGATA12*
	Unnamed__16	Unknown	*MeGATA30*
	Unnamed__2	Unknown	*MeGATA22, MeGATA23, MeGATA31, MeGATA34, MeGATA36*
	Unnamed__4	Unknown	*MeGATA1, MeGATA2, MeGATA3, MeGATA4, MeGATA5, MeGATA6, MeGATA7, MeGATA8, MeGATA9, MeGATA10, MeGATA11, MeGATA12, MeGATA13, MeGATA14, MeGATA15, MeGATA16, MeGATA17, MeGATA18, MeGATA19, MeGATA20, MeGATA21, MeGATA22, MeGATA23, MeGATA24, MeGATA25, MeGATA26, MeGATA27, MeGATA28, MeGATA29, MeGATA30, MeGATA31, MeGATA32, MeGATA33, MeGATA34, MeGATA35, MeGATA36*
	Unnamed__6	Unknown	*MeGATA2, MeGATA5, MeGATA13, MeGATA23, MeGATA24, MeGATA33*
	Unnamed__8	Unknown	*MeGATA12*
	W box	Unknown	*MeGATA3, MeGATA4, MeGATA8, MeGATA10, MeGATA11, MeGATA12, MeGATA13, MeGATA17, MeGATA20, MeGATA23, MeGATA25, MeGATA26, MeGATA30, MeGATA31, MeGATA33, MeGATA35*
	WRE3	Unknown	*MeGATA12, MeGATA13, MeGATA20, MeGATA22, MeGATA24, MeGATA27, MeGATA29, MeGATA30, MeGATA31, MeGATA33*
	Y box	Unknown	*MeGATA34*
