# Supplementary material for: MeGATAs, functional generalists in interactions between cassava growth and development, and abiotic stresses
Source: AoB Plants. 2022 Nov 25;15(1):plac057. doi: 10.1093/aobpla/plac057 (PMC9840210; doi:10.1093/aobpla/plac057)
Supplement: plac057_suppl_Supplementary_Table_S7 [file plac057_suppl_supplementary_table_s7.pdf]

Table S7 The ID number of GATA genes in Arabidopsis and rice

AtGATAs of *Arabidopsis*		*OsGATAs* of rice	
Name	ID	Name	ID
AtGATA1	*At3g24050*	*OSGATA1*	*LOC_Os01g54210*
AtGATA2	*At2g45050*	*OSGATA2*	*LOC_Os02g43150*
AtGATA3	*At4g34680*	*OSGATA3*	*LOC_Os02g56250*
AtGATA4	*At3g60530*	*OSGATA4*	*LOC_Os03g05160*
AtGATA5	*At5g66320*	*OSGATA5*	*LOC_Os04g45650*
AtGATA6	*At3g51080*	*OSGATA6*	*LOC_Os05g44400*
AtGATA7	*At4g36240*	*OSGATA7*	*LOC_Os10g40810*
AtGATA8	*At3g54810*	*OSGATA8*	*LOC_Os01g24070*
AtGATA9	*At4g32890*	*OSGATA9*	*LOC_Os01g47360*
AtGATA10	*At1g08000*	*OSGATA10*	*LOC_Os01g74540*
AtGATA11	*At1g08010*	*OSGATA11*	*LOC_Os02g12790*
AtGATA12	*At5g25830*	*OSGATA12*	*LOC_Os03g61570*
AtGATA13	*At2g28340*	*OSGATA13*	*LOC_Os05g06340*
AtGATA14	*At3g45170*	*OSGATA14*	*LOC_Os05g49280*
AtGATA15	*At3g06740*	*OsGATA15*	*LOC_Os05g50270*
AtGATA16	*At5g49300*	*OSGATA16*	*LOC_Os06g37450*
AtGATA17	*At3g16870*	*OSGATA17*	*LOC_Os02g05510*
AtGATA18	*At3g50870*	*OSGATA18*	*LOC_Os03g47970*
AtGATA19	*At4g36620*	*OSGATA19*	*LOC_Os03g52450*
AtGATA20	*At2g18380*	*OSGATA20*	*LOC_Os06g48534*
AtGATA21	*At5g56860*	*OSGATA21*	*OsJ_34324*
AtGATA22	*At4g26150*	*OSGATA22*	*LOC_Os03g08370*
AtGATA23	*At5g26930*	*OSGATA23*	*LOC_Os07g42400*
AtGATA24	*At3g21175*	*OSGATA24*	*LOC_Os10g32070*
AtGATA25	*At4g24470*	*OSGATA25*	*LOC_Os12g42970*
AtGATA26	*At4g17570*	*OSGATA26*	*LOC_Os12g07120*
AtGATA27	*At5g47140*	*OSGATA27*	*LOC_Os03g03850*
AtGATA28	*At1g51600*	*OSGATA28*	*LOC_Os11g08410*
AtGATA29	*At3g20750*	*OSGATA29*	*LOC_Os04g46020*
AtGATA30	*At4g16141*		
