# Supplementary material for: MeGATAs, functional generalists in interactions between cassava growth and development, and abiotic stresses
Source: AoB Plants. 2022 Nov 25;15(1):plac057. doi: 10.1093/aobpla/plac057 (PMC9840210; doi:10.1093/aobpla/plac057)
Supplement: plac057_suppl_Supplementary_Table_S8 [file plac057_suppl_supplementary_table_s8.pdf]

Table S8 The duplication events of *MeGATA*s

Duplicated pair		Ka	Ks	Ka/Ks	Estimated duplicate time (million year)	Duplicate type
MeGATA2	MeGATA5	0.17	0.59	0.29	19.55	WGD/segmental
MeGATA1	MeGATA4	0.06	0.3	0.19	10.11	WGD/segmental
MeGATA3	MeGATA11	0.39	2.81	0.14	93.65	WGD/segmental
MeGATA3	MeGATA21	0.48	2.7	0.18	89.86	WGD/segmental
MeGATA3	MeGATA24	0.42	2.88	0.15	96.08	WGD/segmental
MeGATA3	MeGATA29	0.42	2.86	0.15	95.44	WGD/segmental
MeGATA6	MeGATA12	0.45	2.75	0.16	91.63	WGD/segmental
MeGATA7	MeGATA8	0.47	2.9	0.16	96.79	WGD/segmental
MeGATA11	MeGATA21	0.68	2.07	0.33	68.86	WGD/segmental
MeGATA11	MeGATA24	0.43	2.88	0.15	96.09	WGD/segmental
MeGATA10	MeGATA30	0.06	0.45	0.14	15.13	WGD/segmental
MeGATA11	MeGATA29	0.06	0.4	0.15	13.42	WGD/segmental
MeGATA8	MeGATA33	0.16	0.41	0.38	13.78	WGD/segmental
MeGATA7	MeGATA33	0.43	2.21	0.2	73.58	WGD/segmental
MeGATA7	MeGATA34	0.12	0.38	0.31	12.57	WGD/segmental
MeGATA9	MeGATA32	0.06	0.57	0.11	18.84	WGD/segmental
MeGATA8	MeGATA34	0.45	2.66	0.17	88.61	WGD/segmental
MeGATA13	MeGATA27	0.07	0.41	0.18	13.78	WGD/segmental
MeGATA13	MeGATA32	0.27	3.37	0.08	112.36	WGD/segmental
MeGATA15	MeGATA26	0.44	2.77	0.16	92.27	WGD/segmental
MeGATA18	MeGATA26	0.11	0.48	0.23	16.07	WGD/segmental
MeGATA20	MeGATA25	0.09	0.41	0.23	13.64	WGD/segmental
MeGATA21	MeGATA24	0.26	0.6	0.44	20.11	WGD/segmental
MeGATA22	MeGATA23	0.11	0.28	0.38	9.36	WGD/segmental
MeGATA21	MeGATA29	0.7	2.08	0.34	69.22	WGD/segmental
MeGATA24	MeGATA29	0.42	3.03	0.14	101.16	WGD/segmental
MeGATA25	MeGATA28	0.54	2.47	0.22	82.38	WGD/segmental
MeGATA27	MeGATA32	0.27	3.37	0.08	112.39	WGD/segmental
MeGATA33	MeGATA34	0.42	1.78	0.23	59.26	WGD/segmental
MeGATA15	MeGATA16	0.44	3.19	0.14	106.19	WGD/segmental
MeGATA17	MeGATA18	0.5	2.62	0.19	87.39	Tandem

Ka, nonsynonymous substitutions rate; Ks, synonymous substitutions rate; WGDs, whole genome duplications
