# Supplementary material for: MeGATAs, functional generalists in interactions between cassava growth and development, and abiotic stresses
Source: AoB Plants. 2022 Nov 25;15(1):plac057. doi: 10.1093/aobpla/plac057 (PMC9840210; doi:10.1093/aobpla/plac057)
Supplement: plac057_suppl_Supplementary_Table_S9 [file plac057_suppl_supplementary_table_s9.pdf]

Table S9 The collinear relationships between *MeGATA*s and GATA genes of other plants

Between cassava and dicots

GATA* pair between cassava and *Arabidopsis

MeGATA		*AtGATA* ID	
**Gene**	**Location**	**Gene**	**Location**
MeGATA28	*Chr11:31120791..31129349 reverse*	AT1G08000.1	Chr1:2483239..2485109 reverse
MeGATA14	*Chr05:4187327..4190046 reverse*	AT2G45050.1	Chr2:18582871..18584083 forward
MeGATA28	*Chr11:31120791..31129349 reverse*	AT2G28340.1	Chr2:12103671..12106172 forward
MeGATA2	*Chr01:32887503..32890589 reverse*	AT3G50870.1	Chr3:18910989..18912608 forward
MeGATA11	*Chr03:31715768..31717300 reverse*	AT3G16870.1	Chr3:5763585..5764654 reverse
MeGATA11	*Chr03:31715768..31717300 reverse*	AT3G06740.1	Chr3:2126413..2127467 forward
MeGATA10	*Ch03:28217689..28222047 reverse*	AT3G24050.1	Chr3:8685872..8687677 forward
MeGATA14	*Chr05:4187327..4190046 reverse*	AT3G60530.1	Chr3:22373214..22374387 forward
MeGATA20	*Chr07:30443131..30446934 forward*	AT3G54810.2	Chr3:20296231..20298691 forward
MeGATA21	*Chr08:35240479..35241728 forward*	AT3G06740.1	Chr3:2126413..2127467 forward
MeGATA24	*Chr09:36650872..36652894 reverse*	AT3G16870.1	Chr3:5763585..5764654 reverse
MeGATA28	*Chr11:31120791..31129349 reverse*	AT3G45170.1	Chr3:16537537..16538305 forward
MeGATA29	*Chr15:718542..720157 forward*	AT3G06740.1	Chr3:2126413..2127467 forward
MeGATA29	*Chr15:718542..720157 forward*	AT3G16870.1	Chr3:5763585..5764654 reverse
MeGATA30	*Chr15:3821573..3825515 reverse*	AT3G24050.1	Chr3:8685872..8687677 forward
MeGATA1	*Chr01:28911239..28918879 forward*	AT4G17570.2	Chr4:9783883..9787528 reverse
MeGATA3	*Chr01:39051943..39053539 reverse*	AT4G16141.1	Chr4:9131649..9133156 reverse
MeGATA6	*Chr02:7725746..7727808 reverse*	AT4G34680.1	Chr4:16553389..16554740 forward
MeGATA5	*Chr02:7375678..7378105 reverse*	AT4G36620.1	Chr4:17268905..17269743 reverse
MeGATA11	*Chr03:31715768..31717300 reverse*	AT4G16141.1	Chr4:9131649..9133156 reverse
MeGATA7	*Chr03:2769834..2771987 reverse*	AT4G26150.1	Chr4:13253209..13254904 forward
MeGATA9	*Chr03:6073458..6075355 reverse*	AT4G32890.1	Chr4:15875469..15876762 forward
MeGATA12	*Chr04:28573452..28575475 reverse*	AT4G34680.1	Chr4:16553389..16554740 forward
MeGATA29	*Chr15:718542..720157 forward*	AT4G16141.1	Chr4:9131649..9133156 reverse
MeGATA32	*Chr16:27768431..27770571 forward*	AT4G32890.1	Chr4:15875469..15876762 forward
MeGATA34	*Chr16:30794414..30797013 forward*	AT4G26150.1	Chr4:13253209..13254904 forward
MeGATA3	*Chr01:39051943..39053539 reverse*	AT5G49300.1	Chr5:19984848..19985697 reverse
MeGATA4	*Chr02:3647601..3658071 forward*	AT5G47140.1	Chr5:19144634..19147489 forward
MeGATA11	*Chr03:31715768..31717300 reverse*	AT5G49300.1	Chr5:19984848..19985697 reverse
MeGATA7	*Chr03:2769834..2771987 reverse*	AT5G56860.1	Chr5:22989264..22991467 reverse
MeGATA13	*Chr04:33357167..33358978 reverse*	AT5G25830.1	Chr5:9004312..9005502 reverse
MeGATA24	*Chr09:36650872..36652894 reverse*	AT5G49300.1	Chr5:19984848..19985697 reverse
MeGATA24	*Chr09:36650872..36652894 reverse*	AT5G26930.1	Chr5:9479465..9480200 forward
MeGATA29	*Chr15:718542..720157 forward*	AT5G49300.1	Chr5:19984848..19985697 reverse
MeGATA33	*Chr16:28568602..28570343 reverse*	AT5G56860.1	Chr5:22989264..22991467 reverse
MeGATA34	*Chr16:30794414..30797013 forward*	AT5G56860.1	Chr5:22989264..22991467 reverse

GATA pair between cassava and poplar

MeGATA		*PtGATA* ID	
**Gene**	**Location**	**Gene**	**Location**
MeGATA1	*Chr01:28911239..28918879 forward*	Potri.001G151700.1	Chr01:12502478..12509652 reverse
MeGATA4	*Chr02:3647601..3658071 forward*	Potri.001G151700.1	Chr01:12502478..12509652 reverse
MeGATA10	*Ch03:28217689..28222047 reverse*	Potri.001G053500.1	Chr01:4086400..4088825 reverse
MeGATA13	*Chr04:33357167..33358978 reverse*	Potri.001G188500.1	Chr01:16658523..16659980 reverse
MeGATA27	*Chro11:3402767..3404317 forward*	Potri.001G188500.1	Chr01:16658523..16659980 reverse
MeGATA30	*Chr15:3821573..3825515 reverse*	Potri.001G053500.1	Chr01:4086400..4088825 reverse
MeGATA32	*Chr16:27768431..27770571 forward*	Potri.001G188500.1	Chr01:16658523..16659980 reverse
MeGATA3	*Chr01:39051943..39053539 reverse*	Potri.002G199800.1	Chr02:16060277..16061389 forward
MeGATA11	*Chr03:31715768..31717300 reverse*	Potri.002G199800.1	Chr02:16060277..16061389 forward
MeGATA15	*Chr05:31189063..31196461 reverse*	Potri.002G110800.1	Chr02:8198185..8203728 reverse
MeGATA14	*Chr05:4187327..4190046 reverse*	Potri.002G142800.1	Chr02:10597459..10598816 forward
MeGATA24	*Chr09:36650872..36652894 reverse*	Potri.002G199800.1	Chr02:16060277..16061389 forward
MeGATA36	*Chr18:4953587..4961297 reverse*	Potri.002G110800.1	Chr02:8198185..8203728 reverse
MeGATA35	*Chr18:4961870..4968617 reverse*	Potri.002G110900.1	Chr02:8206491..8211812 reverse
MeGATA1	*Chr01:28911239..28918879 forward*	Potri.003G082800.1	Chr03:11053998..11060297 forward
MeGATA4	*Chr02:3647601..3658071 forward*	Potri.003G082800.1	Chr03:11053998..11060297 forward
MeGATA10	*Ch03:28217689..28222047 reverse*	Potri.003G174800.1	Chr03:18351163..18353616 forward
MeGATA31	*Chr15:8120914..8123276 reverse*	Potri.003G213300.1	Chr03:21117705..21119661 reverse
MeGATA30	*Chr15:3821573..3825515 reverse*	Potri.003G174800.1	Chr03:18351163..18353616 forward
MeGATA12	*Chr04:28573452..28575475 reverse*	Potri.004G161500.1	Chr04:18221922..18223879 forward
MeGATA28	*Chr11:31120791..31129349 reverse*	Potri.004G211800.1	Chr04:21951640..21956060 reverse
MeGATA11	*Chr03:31715768..31717300 reverse*	Potri.005G020500.1	Chr05:1593396..1595249 forward
MeGATA15	*Chr05:31189063..31196461 reverse*	Potri.005G152800.1	Chr05:14386484..14398559 reverse
MeGATA21	*Chr08:35240479..35241728 forward*	Potri.005G020500.1	Chr05:1593396..1595249 forward
MeGATA24	*Chr09:36650872..36652894 reverse*	Potri.005G020500.1	Chr05:1593396..1595249 forward
MeGATA29	*Chr15:718542..720157 forward*	Potri.005G020500.1	Chr05:1593396..1595249 forward
MeGATA36	*Chr18:4953587..4961297 reverse*	Potri.005G152500.1	Chr05:1593396..1595249 forward
MeGATA35	*Chr18:4961870..4968617 reverse*	Potri.005G152800.1	Chr05:14386484..14398559 reverse
MeGATA8	*Chr03:4349814..4351607 reverse*	Potri.006G229200.1	Chr06:24012189..24015009 forward
MeGATA9	*Chr03:6073458..6075355 reverse*	Potri.006G237700.1	Chr06:24697286..24699200 forward
MeGATA13	*Chr04:33357167..33358978 reverse*	Potri.006G237700.1	Chr06:24697286..24699200 forward
MeGATA27	*Chro11:3402767..3404317 forward*	Potri.006G237700.1	Chr06:24697286..24699200 forward
MeGATA32	*Chr16:27768431..27770571 forward*	Potri.006G237700.1	Chr06:24697286..24699200 forward
MeGATA33	*Chr16:28568602..28570343 reverse*	Potri.006G229200.1	Chr06:24012189..24015009 forward
MeGATA2	*Chr01:32887503..32890589 reverse*	Potri.007G024500.1	Chr07:1849536..1850974 reverse
MeGATA5	*Chr02:7375678..7378105 reverse*	Potri.007G024500.1	Chr07:1849536..1850974 reverse
MeGATA12	*Chr04:28573452..28575475 reverse*	Potri.007G016600.1	Chr07:1263956..1265632 reverse
MeGATA17	*Chr07:4952064..4959681 forward*	Potri.007G116600.1	Chr07:1263956..1265632 reverse
MeGATA18	*Chr07:4939240..4946360 forward*	Potri.007G116500.1	Chr07:13730412..13732003 forward
MeGATA26	*Chr10:24580540..24587376 reverse*	Potri.007G116500.1	Chr07:13730412..13732003 forward
MeGATA11	*Chr03:31715768..31717300 reverse*	Potri.008G213900.1	Chr08:16937467..16938757 reverse
MeGATA25	*Chr10:5104248..5108464 reverse*	Potri.008G038900.1	Chr08:2194429..2198138 forward
MeGATA29	*Chr15:718542..720157 forward*	Potri.008G213900.1	Chr08:16937467..16938757 reverse
MeGATA12	*Chr04:28573452..28575475 reverse*	Potri.009G123400.1	Chr09:10283233..10285131 forward

***GATA* pair between cassava and potato**

MeGATA		*StGATA* ID	
**Gene**	**Location**	**Gene**	***Location***
MeGATA3	*Chr01:39051943..39053539 reverse*	PGSC0003DMP400042956	ST4.03ch01:77915194..77915855 reverse
MeGATA11	*Chr03:31715768..31717300 reverse*	PGSC0003DMP400004168	ST4.03ch01:12242252..12242931 reverse
MeGATA11	*Chr03:31715768..31717300 reverse*	PGSC0003DMP400042956	ST4.03ch01:77915194..77915855 reverse
MeGATA12	*Chr04:28573452..28575475 reverse*	PGSC0003DMP400005648	ST4.03ch01:86628353..86629386 forward
MeGATA14	*Chr05:4187327..4190046 reverse*	PGSC0003DMP400045152	ST4.03ch01:70016091..70016965 forward
MeGATA24	*Chr09:36650872..36652894 reverse*	PGSC0003DMP400042956	ST4.03ch01:77915194..77915855 reverse
MeGATA26	*Chr10:24580540..24587376 reverse*	PGSC0003DMP400056067	ST4.03ch01:82686064..82692543 forward
MeGATA29	*Chr15:718542..720157 forward*	PGSC0003DMP400004168	ST4.03ch01:12242252..12242931 reverse
MeGATA2	*Chr01:32887503..32890589 reverse*	PGSC0003DMP400027172	ST4.03ch02:21158185..21159017 forward
MeGATA5	*Chr02:7375678..7378105 reverse*	PGSC0003DMP400006251	ST4.03ch02:40441585..40442437 reverse
MeGATA12	*Chr04:28573452..28575475 reverse*	PGSC0003DMP400006324	ST4.03ch02:39827894..39829041 reverse
MeGATA13	*Chr04:33357167..33358978 reverse*	PGSC0003DMP400004491	ST4.03ch03:60295522..60296779 reverse
MeGATA27	*Chro11:3402767..3404317 forward*	PGSC0003DMP400004491	ST4.03ch03:60295522..60296779 reverse
MeGATA15	*Chr05:31189063..31196461 reverse*	PGSC0003DMP400016535	ST4.03ch04:65202951..65208104 forward
MeGATA35	*Chr18:4961870..4968617 reverse*	PGSC0003DMP400016535	ST4.03ch04:65202951..65208104 forward
MeGATA11	*Chr03:31715768..31717300 reverse*	PGSC0003DMP400040535	ST4.03ch05:50121041..50122913 reverse
MeGATA20	*Chr07:30443131..30446934 forward*	PGSC0003DMP400040352	ST4.03ch05:51641161..51643349 reverse
MeGATA30	*Chr15:3821573..3825515 reverse*	PGSC0003DMP400047208	ST4.03ch05:48776048..48778242 forward
MeGATA1	*Chr01:28911239..28918879 forward*	PGSC0003DMP400046183	ST4.03ch06:44245546..44252558 forward
MeGATA4	*Chr02:3647601..3658071 forward*	PGSC0003DMP400046183	ST4.03ch06:44245546..44252558 forward
MeGATA25	*Chr10:5104248..5108464 reverse*	PGSC0003DMP400042076	ST4.03ch06:55797390..55801585 forward
MeGATA28	*Chr11:31120791..31129349 reverse*	PGSC0003DMP400042076	ST4.03ch06:55797390..55801585 forward
MeGATA7	*Chr03:2769834..2771987 reverse*	PGSC0003DMP400007959	ST4.03ch07:34660737..34662268 reverse
MeGATA34	*Chr16:30794414..30797013 forward*	PGSC0003DMP400007959	ST4.03ch07:34660737..34662268 reverse
MeGATA1	*Chr01:28911239..28918879 forward*	PGSC0003DMP400035596	ST4.03ch08:1025589..1031616 forward
MeGATA1	*Chr01:28911239..28918879 forward*	PGSC0003DMP400045557	ST4.03ch08:51094613..51101414 reverse
MeGATA4	*Chr02:3647601..3658071 forward*	PGSC0003DMP400035596	ST4.03ch08:1025589..1031616 forward
MeGATA4	*Chr02:3647601..3658071 forward*	PGSC0003DMP400045557	ST4.03ch08:51094613..51101414 reverse
MeGATA9	*Chr03:6073458..6075355 reverse*	PGSC0003DMP400050747	ST4.03ch08:38757143..38758777 forward
MeGATA32	*Chr16:27768431..27770571 forward*	PGSC0003DMP400050747	ST4.03ch08:38757143..38758777 forward
MeGATA3	*Chr01:39051943..39053539 reverse*	PGSC0003DMP400055095	ST4.03ch09:53081711..53082208 reverse
MeGATA11	*Chr03:31715768..31717300 reverse*	PGSC0003DMP400055095	ST4.03ch09:53081711..53082208 reverse
MeGATA10	*Ch03:28217689..28222047 reverse*	PGSC0003DMP400020563	ST4.03ch09:57759755..57761732 forward
MeGATA24	*Chr09:36650872..36652894 reverse*	PGSC0003DMP400055095	ST4.03ch09:53081711..53082208 reverse
MeGATA30	*Chr15:3821573..3825515 reverse*	PGSC0003DMP400020563	ST4.03ch09:57759755..57761732 forward
MeGATA29	*Chr15:718542..720157 forward*	PGSC0003DMP400055095	ST4.03ch09:53081711..53082208 reverse
MeGATA28	*Chr11:31120791..31129349 reverse*	PGSC0003DMP400014220	ST4.03ch11:41571239..41575005 forward
MeGATA7	*Chr03:2769834..2771987 reverse*	PGSC0003DMP400000562	ST4.03ch12:3718235..3720334 forward
MeGATA11	*Chr03:31715768..31717300 reverse*	PGSC0003DMP400008181	ST4.03ch12:60086755..60088071 forward
MeGATA29	*Chr15:718542..720157 forward*	PGSC0003DMP400008181	ST4.03ch12:60086755..60088071 forward
MeGATA34	*Chr16:30794414..30797013 forward*	PGSC0003DMP400000562	ST4.03ch12:3718235..3720334 forward

MeGATA11	*Chr03:31715768..31717300 reverse*	Potri.010G001300.1	Chr10:149902..151135 reverse
MeGATA25	*Chr10:5104248..5108464 reverse*	Potri.010G223300.1	Chr10:20747462..20750870 reverse
MeGATA28	*Chr11:31120791..31129349 reverse*	Potri.010G223300.1	Chr10:20747462..20750870 reverse
MeGATA29	*Chr15:718542..720157 forward*	Potri.010G001300.1	Chr10:149902..151135 reverse
MeGATA3	*Chr01:39051943..39053539 reverse*	Potri.014G124400.1	Chr14:9595038..9596182 forward
MeGATA11	*Chr03:31715768..31717300 reverse*	Potri.014G124400.1	Chr14:9595038..9596182 forward
MeGATA14	*Chr05:4187327..4190046 reverse*	Potri.014G058600.1	Chr14:4540247..4541744 forward
MeGATA24	*Chr09:36650872..36652894 reverse*	Potri.014G124400.1	Chr14:9595038..9596182 forward
MeGATA29	*Chr15:718542..720157 forward*	Potri.014G124400.1	Chr14:9595038..9596182 forward
MeGATA17	*Chr07:4952064..4959681 forward*	Potri.017G042200.1	Chr17:3570411..3576794 reverse
MeGATA26	*Chr10:24580540..24587376 reverse*	Potri.017G042300.1	Chr17:3583592..3583948 reverse
MeGATA9	*Chr03:6073458..6075355 reverse*	Potri.018G044900.1	Chr18:4078771..4080879 reverse
MeGATA8	*Chr03:4349814..4351607*	Potri.018G053600.1	Chr18:5587015..5588879 forward
MeGATA13	*Chr04:33357167..33358978 reverse*	Potri.018G044900.1	Chr18:4078771..4080879 reverse
MeGATA27	*Chro11:3402767..3404317 forward*	Potri.018G044900.1	Chr18:4078771..4080879 reverse
MeGATA34	*Chr16:30794414..30797013 forward*	Potri.018G053600.1	Chr18:5587015..5588879 forward
MeGATA33	*Chr16:28568602..28570343 reverse*	Potri.018G053600.1	Chr18:5587015..5588879 forward
MeGATA32	*Chr16:27768431..27770571 forward*	Potri.018G044900.1	Chr18:4078771..4080879 reverse
MeGATA23	*Chr09:34069727..34071652 reverse*	Potri.019G033000.1	Chr19:3788858..3790666 reverse

Between cassava and monocots

***GATA* pair between cassava and rice**

MeGATA		*OsGATA* ID	
**Gene**	**Location**	**Gene**	**Location**
MeGATA27	*Chro11:3402767..3404317 forward*	LOC_Os01g54210.1	Chr1:31180195..31182456 reverse
MeGATA32	*Chr16:27768431..27770571 forward*	LOC_Os01g54210.1	Chr1:31180195..31182456 reverse
MeGATA14	*Chr05:4187327..4190046 reverse*	LOC_Os10g40810.1	Chr10:21943290..21945186 forward
MeGATA10	*Ch03:28217689..28222047 reverse*	LOC_Os03g05160.1	Chr3:2506063..2507173 forward
MeGATA30	*Chr15:3821573..3825515 reverse*	LOC_Os03g05160.1	Chr3:2506063..2507173 forward
MeGATA1	*Chr01:28911239..28918879 forward*	LOC_Os04g46020.1	Chr4:27260208..27264560 reverse
MeGATA4	*Chr02:3647601..3658071 forward*	LOC_Os04g46020.1	Chr4:27260208..27264560 reverse
MeGATA13	*Chr04:33357167..33358978 reverse*	LOC_Os05g44400.1	Chr5:25832468..25834622 forward
MeGATA24	*Chr09:36650872..36652894 reverse*	LOC_Os05g06340.1	Chr5:3245203..3247503 reverse
MeGATA27	*Chro11:3402767..3404317 forward*	LOC_Os05g44400.1	Chr5:25832468..25834622 forward
MeGATA29	*Chr15:718542..720157 forward*	LOC_Os05g06340.1	Chr5:3245203..3247503 reverse
MeGATA34	*Chr16:30794414..30797013 forward*	LOC_Os06g37450.1	Chr6:22155052..22157061 forward

***GATA* pair between cassava and maize**

MeGATA		*ZmGATA* ID	
**Gene**	**Location**	**Gene**	**Location**
MeGATA27	*Chro11:3402767..3404317 forward*	AC202864.3_FGP002	Chr6:160741348-160742841
MeGATA27	*Chro11:3402767..3404317 forward*	GRMZM2G135381_P01	Chr8:123887193-123889333
MeGATA14	*Chr05:4187327..4190046 reverse*	GRMZM2G101058_P01	Chr9:115980192-115982001

***GATA* pair between cassava and sorghum**

MeGATA		*SbGATA* ID	
**Gene**	**Location**	**Gene**	**Location**
MeGATA14	*Chr05:4187327..4190046 reverse*	Sobic.001G299600	Chr01:58059100..58064037 forward
MeGATA30	*Chr15:3821573..3825515 reverse*	Sobic.001G229200	Chr01:22034007..22036965 forward
MeGATA30	*Chr15:3821573..3825515 reverse*	Sobic.001G506066	Chr01:77412195..77414262 reverse
MeGATA11	*Chr03:31715768..31717300 reverse*	Sobic.003G157300	Chr03:17586415..17589199 forward
MeGATA24	*Chr09:36650872..36652894 reverse*	Sobic.003G157300	Chr03:17586415..17589199 forward
MeGATA29	*Chr15:718542..720157 forward*	Sobic.003G157300	Chr03:17586415..17589199 forward
MeGATA32	*Chr16:27768431..27770571 forward*	Sobic.003G293100	Chr03:62553926..62556698 reverse
MeGATA1	*Chr01:28911239..28918879 forward*	Sobic.006G166100	Chr06:52353441..52359462 reverse
MeGATA4	*Chr02:3647601..3658071 forward*	Sobic.006G166100	Chr06:52353441..52359462 reverse
MeGATA13	*Chr04:33357167..33358978 reverse*	Sobic.009G202000	Chr09:55131728..55133587 forward
MeGATA24	*Chr09:36650872..36652894 reverse*	Sobic.009G050600	Chr09:5030455..5032765 reverse
MeGATA27	*Chro11:3402767..3404317 forward*	Sobic.009G202000	Chr09:55131728..55133587 forward
MeGATA29	*Chr15:718542..720157 forward*	Sobic.009G050600	Chr09:5030455..5032765 reverse
MeGATA34	*Chr16:30794414..30797013 forward*	Sobic.010G173400	Chr10:50865038..50868077 forward
