# Supplementary material for: MeGATAs, functional generalists in interactions between cassava growth and development, and abiotic stresses
Source: AoB Plants. 2022 Nov 25;15(1):plac057. doi: 10.1093/aobpla/plac057 (PMC9840210; doi:10.1093/aobpla/plac057)
Supplement: plac057_suppl_Supplementary_Table_S10 [file plac057_suppl_supplementary_table_s10.pdf]

Table S10 The cassava genes potentially targeted by MeGATAs

MeGATA	Target gene
MeGATA10	Manes.01G054300
MeGATA10	Manes.01G056200
MeGATA10	Manes.01G131300
MeGATA10	Manes.01G132000
MeGATA10	Manes.01G144900
MeGATA10	Manes.01G163300
MeGATA10	Manes.01G178100
MeGATA10	Manes.01G190700
MeGATA10	Manes.01G199000
MeGATA10	Manes.01G214800
MeGATA10	Manes.01G217200
MeGATA10	Manes.01G222100
MeGATA10	Manes.01G268800
MeGATA10	Manes.02G001700
MeGATA10	Manes.02G015100
MeGATA10	Manes.02G060100
MeGATA10	Manes.02G090700
MeGATA10	Manes.02G118100
MeGATA10	Manes.02G138100
MeGATA10	Manes.02G139300
MeGATA10	Manes.02G155400
MeGATA10	Manes.02G170100
MeGATA10	Manes.02G180600
MeGATA10	Manes.02G184100
MeGATA10	Manes.02G202200
MeGATA10	Manes.02G211000
MeGATA10	Manes.03G011400
MeGATA10	Manes.03G026500
MeGATA10	Manes.03G035400
MeGATA10	Manes.03G075500
MeGATA10	Manes.03G077200
MeGATA10	Manes.03G094500
MeGATA10	Manes.03G100500
MeGATA10	Manes.03G110800
MeGATA10	Manes.03G122600
MeGATA10	Manes.03G133800
MeGATA10	Manes.03G170300
MeGATA10	Manes.04G007800
MeGATA10	Manes.04G020900
MeGATA10	Manes.04G021800
MeGATA10	Manes.04G099100
MeGATA10	Manes.04G116400
MeGATA10	Manes.04G117100
MeGATA10	Manes.04G132700
MeGATA10	Manes.04G138400
MeGATA10	Manes.04G154100
MeGATA10	Manes.04G158200
MeGATA10	Manes.05G013900
MeGATA10	Manes.05G018600
MeGATA10	Manes.05G028900
MeGATA10	Manes.05G040400
MeGATA10	Manes.05G045000
MeGATA10	Manes.05G066700
MeGATA10	Manes.05G077100
MeGATA10	Manes.05G107900
MeGATA10	Manes.05G149600
MeGATA10	Manes.05G156800
MeGATA10	Manes.05G171800
MeGATA10	Manes.05G175800
MeGATA10	Manes.05G185800
MeGATA10	Manes.06G026900
MeGATA10	Manes.06G079600
MeGATA10	Manes.06G111400
MeGATA10	Manes.06G118500
MeGATA10	Manes.06G121300
MeGATA10	Manes.06G162100
MeGATA10	Manes.07G036400
MeGATA10	Manes.07G097400
MeGATA10	Manes.07G102200
MeGATA10	Manes.07G130100
MeGATA10	Manes.07G138700
MeGATA10	Manes.08G014500

MeGATA10	Manes.08G024100
MeGATA10	Manes.08G087000
MeGATA10	Manes.08G174400
MeGATA10	Manes.09G011100
MeGATA10	Manes.09G014600
MeGATA10	Manes.09G031300
MeGATA10	Manes.09G032500
MeGATA10	Manes.09G032600
MeGATA10	Manes.09G038600
MeGATA10	Manes.09G045600
MeGATA10	Manes.09G056600
MeGATA10	Manes.09G063900
MeGATA10	Manes.09G114600
MeGATA10	Manes.09G116600
MeGATA10	Manes.09G141600
MeGATA10	Manes.09G151400
MeGATA10	Manes.09G171900
MeGATA10	Manes.09G172800
MeGATA10	Manes.09G176000
MeGATA10	Manes.10G015200
MeGATA10	Manes.10G016900
MeGATA10	Manes.10G042600
MeGATA10	Manes.10G086500
MeGATA10	Manes.10G101700
MeGATA10	Manes.10G109500
MeGATA10	Manes.10G129800
MeGATA10	Manes.11G028100
MeGATA10	Manes.11G040100
MeGATA10	Manes.11G041300
MeGATA10	Manes.11G061800
MeGATA10	Manes.11G097200
MeGATA10	Manes.11G127000
MeGATA10	Manes.11G150800
MeGATA10	Manes.12G022100
MeGATA10	Manes.12G036700
MeGATA10	Manes.12G065900
MeGATA10	Manes.12G077200
MeGATA10	Manes.12G084800
MeGATA10	Manes.12G103400
MeGATA10	Manes.12G114100
MeGATA10	Manes.12G133500
MeGATA10	Manes.12G148400
MeGATA10	Manes.12G153900
MeGATA10	Manes.13G014300
MeGATA10	Manes.13G020000
MeGATA10	Manes.13G023200
MeGATA10	Manes.13G024800
MeGATA10	Manes.13G024900
MeGATA10	Manes.13G042800
MeGATA10	Manes.13G096100
MeGATA10	Manes.13G111600
MeGATA10	Manes.13G123700
MeGATA10	Manes.13G135500
MeGATA10	Manes.13G148800
MeGATA10	Manes.14G018500
MeGATA10	Manes.14G028200
MeGATA10	Manes.14G035100
MeGATA10	Manes.14G089300
MeGATA10	Manes.14G136900
MeGATA10	Manes.14G138800
MeGATA10	Manes.14G140100
MeGATA10	Manes.14G141900
MeGATA10	Manes.14G166000
MeGATA10	Manes.15G070600
MeGATA10	Manes.15G091000
MeGATA10	Manes.15G092400
MeGATA10	Manes.15G120000
MeGATA10	Manes.15G142100
MeGATA10	Manes.15G163500
MeGATA10	Manes.15G168200
MeGATA10	Manes.15G177600
MeGATA10	Manes.16G046700
MeGATA10	Manes.16G048400
MeGATA10	Manes.16G052100
MeGATA10	Manes.16G063000
MeGATA10	Manes.17G012200
MeGATA10	Manes.17G029000

MeGATA10	Manes.18G029800
MeGATA10	Manes.18G046800
MeGATA10	Manes.18G053700
MeGATA10	Manes.18G069600
MeGATA10	Manes.18G075500
MeGATA10	Manes.18G114700
MeGATA10	Manes.18G118000
MeGATA10	Manes.S007700
MeGATA10	Manes.S020000
MeGATA10	Manes.S022900
MeGATA10	Manes.S027500
MeGATA10	Manes.S052700
MeGATA10	Manes.S072000
MeGATA16	Manes.01G163700
MeGATA16	Manes.01G170200
MeGATA16	Manes.01G235300
MeGATA16	Manes.02G039400
MeGATA16	Manes.02G056500
MeGATA16	Manes.02G149900
MeGATA16	Manes.02G155100
MeGATA16	Manes.02G161300
MeGATA16	Manes.03G062000
MeGATA16	Manes.03G086700
MeGATA16	Manes.03G121600
MeGATA16	Manes.03G160600
MeGATA16	Manes.03G206000
MeGATA16	Manes.04G124600
MeGATA16	Manes.05G047000
MeGATA16	Manes.05G097000
MeGATA16	Manes.05G108900
MeGATA16	Manes.06G017200
MeGATA16	Manes.06G116700
MeGATA16	Manes.06G168000
MeGATA16	Manes.07G116000
MeGATA16	Manes.08G031200
MeGATA16	Manes.08G037800
MeGATA16	Manes.08G051800
MeGATA16	Manes.08G078600
MeGATA16	Manes.08G091400
MeGATA16	Manes.08G148200
MeGATA16	Manes.09G060000
MeGATA16	Manes.09G140800
MeGATA16	Manes.10G031400
MeGATA16	Manes.10G094400
MeGATA16	Manes.10G118200
MeGATA16	Manes.11G082600
MeGATA16	Manes.11G131400
MeGATA16	Manes.11G133700
MeGATA16	Manes.12G009700
MeGATA16	Manes.12G083000
MeGATA16	Manes.12G110600
MeGATA16	Manes.13G113000
MeGATA16	Manes.13G144200
MeGATA16	Manes.13G151600
MeGATA16	Manes.13G151700
MeGATA16	Manes.15G026500
MeGATA16	Manes.15G036800
MeGATA16	Manes.15G044100
MeGATA16	Manes.15G075400
MeGATA16	Manes.15G110300
MeGATA16	Manes.15G117300
MeGATA16	Manes.16G044100
MeGATA16	Manes.16G096000
MeGATA16	Manes.16G134800
MeGATA16	Manes.17G065100
MeGATA16	Manes.S023700
MeGATA16	Manes.S075400
MeGATA18	Manes.01G001900
MeGATA18	Manes.01G053500
MeGATA18	Manes.01G054300
MeGATA18	Manes.01G056200
MeGATA18	Manes.01G057700
MeGATA18	Manes.01G061500
MeGATA18	Manes.01G073000
MeGATA18	Manes.01G082500
MeGATA18	Manes.01G156900
MeGATA18	Manes.01G176000

MeGATA18	Manes.02G001700
MeGATA18	Manes.02G002300
MeGATA18	Manes.02G002400
MeGATA18	Manes.02G118100
MeGATA18	Manes.02G139300
MeGATA18	Manes.02G161700
MeGATA18	Manes.02G211000
MeGATA18	Manes.03G006800
MeGATA18	Manes.03G110800
MeGATA18	Manes.03G133900
MeGATA18	Manes.03G180800
MeGATA18	Manes.04G006800
MeGATA18	Manes.04G076400
MeGATA18	Manes.04G125000
MeGATA18	Manes.04G133500
MeGATA18	Manes.04G147800
MeGATA18	Manes.05G098900
MeGATA18	Manes.05G173900
MeGATA18	Manes.05G185800
MeGATA18	Manes.06G089900
MeGATA18	Manes.06G121300
MeGATA18	Manes.07G001400
MeGATA18	Manes.07G011300
MeGATA18	Manes.07G065200
MeGATA18	Manes.07G093500
MeGATA18	Manes.07G117000
MeGATA18	Manes.07G120100
MeGATA18	Manes.08G006700
MeGATA18	Manes.08G024100
MeGATA18	Manes.08G033100
MeGATA18	Manes.08G115200
MeGATA18	Manes.09G032500
MeGATA18	Manes.09G032600
MeGATA18	Manes.09G045600
MeGATA18	Manes.09G056600
MeGATA18	Manes.09G116600
MeGATA18	Manes.10G109500
MeGATA18	Manes.11G040100
MeGATA18	Manes.11G041300
MeGATA18	Manes.11G072800
MeGATA18	Manes.11G097200
MeGATA18	Manes.11G150800
MeGATA18	Manes.12G004700
MeGATA18	Manes.12G065900
MeGATA18	Manes.12G103400
MeGATA18	Manes.12G123600
MeGATA18	Manes.12G148400
MeGATA18	Manes.13G068200
MeGATA18	Manes.13G085000
MeGATA18	Manes.13G085200
MeGATA18	Manes.13G123700
MeGATA18	Manes.13G135500
MeGATA18	Manes.13G145200
MeGATA18	Manes.14G034000
MeGATA18	Manes.14G044900
MeGATA18	Manes.14G089300
MeGATA18	Manes.14G116800
MeGATA18	Manes.14G136900
MeGATA18	Manes.15G000200
MeGATA18	Manes.15G019800
MeGATA18	Manes.15G028200
MeGATA18	Manes.15G091000
MeGATA18	Manes.15G142100
MeGATA18	Manes.15G162000
MeGATA18	Manes.16G046700
MeGATA18	Manes.16G052100
MeGATA18	Manes.16G057200
MeGATA18	Manes.16G080500
MeGATA18	Manes.17G048400
MeGATA18	Manes.17G082400
MeGATA18	Manes.17G109100
MeGATA18	Manes.18G003600
MeGATA18	Manes.18G046800
MeGATA18	Manes.18G081200
MeGATA18	Manes.18G141700
MeGATA18	Manes.S013700
MeGATA18	Manes.S044000

MeGATA18	Manes.S076100
MeGATA28	Manes.01G245600
MeGATA28	Manes.02G199500
MeGATA28	Manes.03G061800
MeGATA28	Manes.04G115500
MeGATA28	Manes.05G108900
MeGATA28	Manes.06G155000
MeGATA28	Manes.07G015400
MeGATA28	Manes.07G125900
MeGATA28	Manes.10G002000
MeGATA28	Manes.11G095800
MeGATA28	Manes.11G127500
MeGATA28	Manes.14G042600
MeGATA28	Manes.18G028400
MeGATA29	Manes.01G074600
MeGATA29	Manes.01G176000
MeGATA29	Manes.02G142400
MeGATA29	Manes.02G161700
MeGATA29	Manes.02G211000
MeGATA29	Manes.03G186600
MeGATA29	Manes.04G098500
MeGATA29	Manes.04G125000
MeGATA29	Manes.07G065200
MeGATA29	Manes.08G033100
MeGATA29	Manes.09G032500
MeGATA29	Manes.09G032600
MeGATA29	Manes.09G045600
MeGATA29	Manes.10G109500
MeGATA29	Manes.11G029700
MeGATA29	Manes.11G040100
MeGATA29	Manes.11G097200
MeGATA29	Manes.12G004700
MeGATA29	Manes.12G103400
MeGATA29	Manes.12G148400
MeGATA29	Manes.13G085000
MeGATA29	Manes.13G085200
MeGATA29	Manes.13G123700
MeGATA29	Manes.15G091000
MeGATA29	Manes.16G046700
MeGATA29	Manes.16G080500
MeGATA29	Manes.17G048400
MeGATA29	Manes.18G046800
MeGATA29	Manes.S076100
MeGATA32	Manes.01G245600
MeGATA32	Manes.02G102800
MeGATA32	Manes.04G058700
MeGATA32	Manes.04G115500
MeGATA32	Manes.05G108900
MeGATA32	Manes.06G155000
MeGATA32	Manes.11G095800
MeGATA32	Manes.18G028400
MeGATA32	Manes.18G139600
