# Supplementary material for: MeGATAs, functional generalists in interactions between cassava growth and development, and abiotic stresses
Source: AoB Plants. 2022 Nov 25;15(1):plac057. doi: 10.1093/aobpla/plac057 (PMC9840210; doi:10.1093/aobpla/plac057)
Supplement: plac057_suppl_Supplementary_Table_S11 [file plac057_suppl_supplementary_table_s11.pdf]

Table S11 Information on miRNAs and miRNAs-acted *MeGATA*s

miRNA_Acc.	Target	Expectation	UPE$	miRNA_start	miRNA_end	Target_start	Target_end	miRNA_aligned_fragment	Alignment	Target_aligned_fragment	Inhibition	Target_ Desc.	Multiplicity
mes-miR159c	MeGATA23	2.5	-1	1	21	1041	1061	AUUGGAGUGAAGGGAGCUCUG	::::: ::: :::::::::	GUGAGCUACCUGCACUCCAAU	Translation		2
mes-miR159d	MeGATA23	2.5	-1	1	21	1041	1061	AUUGGAGUGAAGGGAGCUCUG	::::: ::: :::::::::	GUGAGCUACCUGCACUCCAAU	Translation		2
mes-miR159c	MeGATA22	3	-1	1	21	1026	1046	AUUGGAGUGAAGGGAGCUCUG	::::: ::: ::.::::::	GUGAGCUACCUGCAUUCCAAU	Translation		2
mes-miR159d	MeGATA22	3	-1	1	21	1026	1046	AUUGGAGUGAAGGGAGCUCUG	::::: ::: ::.::::::	GUGAGCUACCUGCAUUCCAAU	Translation		2
mes-miR156h	MeGATA28	4	-1	1	21	1297	1317	UUGACAGAAGAUAGAGAGCAC	::.:::::::.::::::.	AUGUUCUCUAUUUUCUGUUUU	Cleavage		1
mes-miR156i	MeGATA28	4	-1	1	21	1297	1317	UUGACAGAAGAUAGAGAGCAC	::.:::::::.::::::.	AUGUUCUCUAUUUUCUGUUUU	Cleavage		1
mes-miR156j	MeGATA28	4	-1	1	21	1297	1317	UUGACAGAAGAUAGAGAGCAC	::.:::::::.::::::.	AUGUUCUCUAUUUUCUGUUUU	Cleavage		1
mes-miR156k	MeGATA28	4	-1	1	21	1296	1316	UGACAGAAGAGAGAGAGCACA	: ::.:::: :.::::::.	UAUGUUCUCUAUUUUCUGUUU	Translation		1
mes-miR156k	MeGATA6	4.5	-1	1	21	1339	1359	UGACAGAAGAGAGAGAGCACA	: : .:.:.:.:.::.:::.:	UUUUUUUUUUUUUUUUUGUUA	Cleavage		1
mes-miR159c	MeGATA23	4.5	-1	1	21	664	684	AUUGGAGUGAAGGGAGCUCUG	:::: : .::::..::::::	CAGAUCCUCCUUUGCUCCAAC	Cleavage		2
mes-miR159c	MeGATA22	4.5	-1	1	21	628	648	AUUGGAGUGAAGGGAGCUCUG	:::: : .::::..::::::	CAGACCCUCCUUUGCUCCAAC	Cleavage		2
mes-miR159d	MeGATA23	4.5	-1	1	21	664	684	AUUGGAGUGAAGGGAGCUCUG	:::: : .::::..::::::	CAGAUCCUCCUUUGCUCCAAC	Cleavage		2
mes-miR159d	MeGATA22	4.5	-1	1	21	628	648	AUUGGAGUGAAGGGAGCUCUG	:::: : .::::..::::::	CAGACCCUCCUUUGCUCCAAC	Cleavage		2
mes-miR319a	MeGATA4	4.5	-1	1	21	1895	1915	UUGGACUGAAGGGAGCUCCCU	: :: ::. :::.:::::::	AUGGCUCUUGCUUUAGUCCAA	Cleavage		1
mes-miR319b	MeGATA4	4.5	-1	1	21	1895	1915	UUGGACUGAAGGGAGCUCCCU	: :: ::. :::.:::::::	AUGGCUCUUGCUUUAGUCCAA	Cleavage		1
mes-miR319c	MeGATA4	4.5	-1	1	21	1895	1915	UUGGACUGAAGGGAGCUCCCU	: :: ::. :::.:::::::	AUGGCUCUUGCUUUAGUCCAA	Cleavage		1
mes-miR319d	MeGATA4	4.5	-1	1	21	1895	1915	UUGGACUGAAGGGAGCUCCCU	: :: ::. :::.:::::::	AUGGCUCUUGCUUUAGUCCAA	Cleavage		1
mes-miR319e	MeGATA4	4.5	-1	1	21	1895	1915	UUGGACUGAAGGGAGCUCCCU	: :: ::. :::.:::::::	AUGGCUCUUGCUUUAGUCCAA	Cleavage		1
mes-miR319f	MeGATA4	4.5	-1	1	21	1895	1915	UUGGACUGAAGGGAGCUCCUU	: :: ::. :::.:::::::	AUGGCUCUUGCUUUAGUCCAA	Cleavage		1
mes-miR319g	MeGATA4	4.5	-1	1	21	1895	1915	UUGGACUGAAGGGAGCUCCUU	: :: ::. :::.:::::::	AUGGCUCUUGCUUUAGUCCAA	Cleavage		1
mes-miR319h	MeGATA4	4.5	-1	1	21	1896	1916	CUUGGACUGAAGGGAGCUCCU	:: ::. :::.::::::::	UGGCUCUUGCUUUAGUCCAAG	Cleavage		1
mes-miR397	MeGATA10	4.5	-1	1	20	1855	1874	UUUGAGUGCAGCGUUGAUGA	:.:: : .:.::.:.::::	UUAUAACUGUUGUAUUCAAA	Cleavage		1
mes-miR399e	MeGATA25	4.5	-1	1	21	290	310	UGCCAAAGGAGAUUUGCUCGG	::: :::.:.:::::: :	UGGAGAUAAUUUUCUUUGGAA	Cleavage		1
mes-miR156a	MeGATA28	5	-1	1	20	1297	1316	UGACAGAAGAGAGUGAGCAC	::.:: :: :.::::::.	AUGUUCUCUAUUUUCUGUUU	Translation		1
mes-miR156b	MeGATA28	5	-1	1	20	1297	1316	UGACAGAAGAGAGUGAGCAC	::.:: :: :.::::::.	AUGUUCUCUAUUUUCUGUUU	Translation		1
mes-miR156c	MeGATA28	5	-1	1	20	1297	1316	UGACAGAAGAGAGUGAGCAC	::.:: :: :.::::::.	AUGUUCUCUAUUUUCUGUUU	Translation		1
mes-miR156d	MeGATA28	5	-1	1	20	1297	1316	UGACAGAAGAGAGUGAGCAC	::.:: :: :.::::::.	AUGUUCUCUAUUUUCUGUUU	Translation		1
mes-miR156e	MeGATA28	5	-1	1	20	1297	1316	UGACAGAAGAGAGUGAGCAC	::.:: :: :.::::::.	AUGUUCUCUAUUUUCUGUUU	Translation		1
mes-miR156f	MeGATA28	5	-1	1	20	1297	1316	UGACAGAAGAGAGUGAGCAC	::.:: :: :.::::::.	AUGUUCUCUAUUUUCUGUUU	Translation		1
mes-miR156g	MeGATA28	5	-1	1	20	1297	1316	UGACAGAAGAGAGUGAGCAC	::.:: :: :.::::::.	AUGUUCUCUAUUUUCUGUUU	Translation		1
mes-miR156k	MeGATA14	5	-1	1	21	1204	1224	UGACAGAAGAGAGAGAGCACA	::.:.:.:.:.::.: :.:	GCUGUUUUUUUUUUUUUUUUA	Cleavage		1
mes-miR156k	MeGATA18	5	-1	1	21	441	461	UGACAGAAGAGAGAGAGCACA	: : ::.:::.: :::::: :	UAUUCUUUCUUUAUUCUGUGA	Cleavage		2
mes-miR156k	MeGATA18	5	-1	1	21	472	492	UGACAGAAGAGAGAGAGCACA	: .:.:.:.:.::::::.	AUUCUUUUUUUUUUUCUGUUU	Cleavage		2
mes-miR156k	MeGATA32	5	-1	1	21	37	57	UGACAGAAGAGAGAGAGCACA	: ::::: :::::: ::	ACUUCUCUCACUCUUCUCUCU	Cleavage		1
mes-miR159a	MeGATA23	5	-1	1	21	1041	1061	UUUGGAUUGAAGGGAGCUCUA	::::: ::: :: :::::	GUGAGCUACCUGCACUCCAAU	Translation		1
mes-miR159a	MeGATA22	5	-1	1	21	1026	1046	UUUGGAUUGAAGGGAGCUCUA	::::: ::: :: :::::	GUGAGCUACCUGCAUUCCAAU	Translation		1
mes-miR159b	MeGATA23	5	-1	1	21	1041	1061	UUUGGAUUGAAGGGAGCUCUA	::::: ::: :: :::::	GUGAGCUACCUGCACUCCAAU	Translation		1
mes-miR159b	MeGATA22	5	-1	1	21	1026	1046	UUUGGAUUGAAGGGAGCUCUA	::::: ::: :: :::::	GUGAGCUACCUGCAUUCCAAU	Translation		1
mes-miR164a	MeGATA6	5	-1	1	21	72	92	UGGAGAAGCAGGGCACGUGCA	::: : :..::.::::.:	ACCACCUCCUUUGUUUCUUCU	Cleavage		1
mes-miR164a	MeGATA8	5	-1	1	21	131	151	UGGAGAAGCAGGGCACGUGCA	::: : ..:: :::.::::	CCCACUUCUUCUUCUUUUCCA	Cleavage		1
mes-miR164b	MeGATA6	5	-1	1	21	72	92	UGGAGAAGCAGGGCACGUGCA	::: : :..::.::::.:	ACCACCUCCUUUGUUUCUUCU	Cleavage		1
mes-miR164b	MeGATA8	5	-1	1	21	131	151	UGGAGAAGCAGGGCACGUGCA	::: : ..:: :::.::::	CCCACUUCUUCUUCUUUUCCA	Cleavage		1
mes-miR164c	MeGATA6	5	-1	1	21	72	92	UGGAGAAGCAGGGCACGUGCA	::: : :..::.::::.:	ACCACCUCCUUUGUUUCUUCU	Cleavage		1
mes-miR164c	MeGATA8	5	-1	1	21	131	151	UGGAGAAGCAGGGCACGUGCA	::: : ..:: :::.::::	CCCACUUCUUCUUCUUUUCCA	Cleavage		1
mes-miR167a	MeGATA18	5	-1	1	21	1185	1205	UGAAGCUGCCAGCAUGAUCUG	: : :::::::: : :::::	GAUAGCAUGCUGGAAACUUCA	Cleavage		1
mes-miR167b	MeGATA18	5	-1	1	21	1185	1205	UGAAGCUGCCAGCAUGAUCUA	: : :::::::: : :::::	GAUAGCAUGCUGGAAACUUCA	Cleavage		1
mes-miR167c	MeGATA18	5	-1	1	21	1185	1205	UGAAGCUGCCAGCAUGAUCUA	: : :::::::: : :::::	GAUAGCAUGCUGGAAACUUCA	Cleavage		1
mes-miR167d	MeGATA18	5	-1	1	22	1184	1205	UGAAGCUGCCAGCAUGAUCUGA	: : : :::::::: : :::::	UGAUAGCAUGCUGGAAACUUCA	Cleavage		1
mes-miR167e	MeGATA18	5	-1	1	22	1184	1205	UGAAGCUGCCAGCAUGAUCUGA	: : : :::::::: : :::::	UGAUAGCAUGCUGGAAACUUCA	Cleavage		1
mes-miR167f	MeGATA18	5	-1	1	22	1184	1205	UGAAGCUGCCAGCAUGAUCUGA	: : : :::::::: : :::::	UGAUAGCAUGCUGGAAACUUCA	Cleavage		1
mes-miR167g	MeGATA18	5	-1	1	21	1185	1205	UGAAGCUGCCAGCAUGAUCUU	.: : :::::::: : :::::	GAUAGCAUGCUGGAAACUUCA	Cleavage		1
mes-miR167h	MeGATA18	5	-1	1	21	1185	1205	UGAAGCUGCCAGCAUGAUCUU	.: : :::::::: : :::::	GAUAGCAUGCUGGAAACUUCA	Cleavage		1
mes-miR171a	MeGATA26	5	-1	1	21	122	142	GGAUUGAGCCGCGUCAAUAUC	:: : ::.::: ::::::	ACUAAUUCCGUGGCACAAUCC	Cleavage		1
mes-miR171g	MeGATA29	5	-1	1	21	160	180	UGAUUGAGCCGUGCCAAUAUC	.:.: ::. :::.::.::.:	UGUGUCGGUUCGGUUCGAUUA	Cleavage		1
mes-miR171h	MeGATA29	5	-1	1	21	160	180	UGAUUGAGCCGUGCCAAUAUC	.:.: ::. :::.::.::.:	UGUGUCGGUUCGGUUCGAUUA	Cleavage		1
mes-miR171i	MeGATA29	5	-1	1	21	160	180	UGAUUGAGCCGUGCCAAUAUC	.:.: ::. :::.::.::.:	UGUGUCGGUUCGGUUCGAUUA	Cleavage		1
mes-miR171j	MeGATA29	5	-1	1	21	160	180	UGAUUGAGCCGUGCCAAUAUC	.:.: ::. :::.::.::.:	UGUGUCGGUUCGGUUCGAUUA	Cleavage		1
mes-miR171k	MeGATA29	5	-1	1	21	160	180	UGAUUGAGCCGUGCCAAUAUC	.:.: ::. :::.::.::.:	UGUGUCGGUUCGGUUCGAUUA	Cleavage		1
mes-miR172a	MeGATA15	5	-1	1	21	277	297	AGAAUCUUGAUGAUGCUGCAU	::::. :.:.:::.::.:	GACCAGCGACGUUAAGGUUUU	Cleavage		1
mes-miR172a	MeGATA36	5	-1	1	21	1198	1218	AGAAUCUUGAUGAUGCUGCAU	:::: :.: ::::.::.:	UCCCAGCCUUAACAAGGUUUU	Translation		1

mes-miR172b	MeGATA15	5	-1	1	21	277	297	AGAAUCUUGAUGAUGCUGCAU	::::. :.:.:::.::.:	GACCAGCGACGUUAAGGUUUU	Cleavage	1
mes-miR172b	MeGATA36	5	-1	1	21	1198	1218	AGAAUCUUGAUGAUGCUGCAU	:::: :.: ::::.::.:	UCCCAGCCUUAACAAGGUUUU	Translation	1
mes-miR172d	MeGATA15	5	-1	1	21	277	297	AGAAUCUUGAUGAUGCUGCAU	::::. :.:.:::.::.:	GACCAGCGACGUUAAGGUUUU	Cleavage	1
mes-miR172d	MeGATA36	5	-1	1	21	1198	1218	AGAAUCUUGAUGAUGCUGCAU	:::: :.: ::::.::.:	UCCCAGCCUUAACAAGGUUUU	Translation	1
mes-miR319a	MeGATA1	5	-1	1	21	1930	1950	UUGGACUGAAGGGAGCUCCCU	: :: ::. :::.:::::.:	AUGGCUCUUGCUUUAGUCCGA	Cleavage	1
mes-miR319b	MeGATA1	5	-1	1	21	1930	1950	UUGGACUGAAGGGAGCUCCCU	: :: ::. :::.:::::.:	AUGGCUCUUGCUUUAGUCCGA	Cleavage	1
mes-miR319c	MeGATA1	5	-1	1	21	1930	1950	UUGGACUGAAGGGAGCUCCCU	: :: ::. :::.:::::.:	AUGGCUCUUGCUUUAGUCCGA	Cleavage	1
mes-miR319d	MeGATA1	5	-1	1	21	1930	1950	UUGGACUGAAGGGAGCUCCCU	: :: ::. :::.:::::.:	AUGGCUCUUGCUUUAGUCCGA	Cleavage	1
mes-miR319e	MeGATA1	5	-1	1	21	1930	1950	UUGGACUGAAGGGAGCUCCCU	: :: ::. :::.:::::.:	AUGGCUCUUGCUUUAGUCCGA	Cleavage	1
mes-miR319f	MeGATA1	5	-1	1	21	1930	1950	UUGGACUGAAGGGAGCUCCUU	: :: ::. :::.:::::.:	AUGGCUCUUGCUUUAGUCCGA	Cleavage	1
mes-miR319g	MeGATA1	5	-1	1	21	1930	1950	UUGGACUGAAGGGAGCUCCUU	: :: ::. :::.:::::.:	AUGGCUCUUGCUUUAGUCCGA	Cleavage	1
mes-miR319h	MeGATA1	5	-1	1	21	1931	1951	CUUGGACUGAAGGGAGCUCCU	:: ::. :::.:::::.::	UGGCUCUUGCUUUAGUCCGAG	Cleavage	1
mes-miR319h	MeGATA23	5	-1	1	21	1041	1061	CUUGGACUGAAGGGAGCUCCU	::::: ::: :: :::::	GUGAGCUACCUGCACUCCAAU	Translation	1
mes-miR319h	MeGATA22	5	-1	1	21	1026	1046	CUUGGACUGAAGGGAGCUCCU	::::: ::: :: :::::	GUGAGCUACCUGCAUUCCAAU	Translation	1
mes-miR319h	MeGATA12	5	-1	1	21	1128	1148	CUUGGACUGAAGGGAGCUCCU	:: :: ::: .::::::	UAGACCUGCCUGUAGUCCAAC	Translation	1
mes-miR390	MeGATA6	5	-1	1	20	114	132	CGCUAUCCAUCCUGAGUUUC	:.:::: :::::::::	CAGACUCAA-AUGGAUAGCG	Translation	1
mes-miR395e	MeGATA25	5	-1	1	21	1091	1111	CUGAAGGGUUUGGAGGAACUC	:::::: ..:..:::: :.:	CAGUUCCAUUAGGCCCUGCGG	Cleavage	1
mes-miR396a	MeGATA23	5	-1	1	21	1062	1082	UUCCACAGCUUUCUUGAACUG	::::::::.: :::::	UCUCACAAGAAAGUUAUGGAA	Cleavage	1
mes-miR396a	MeGATA22	5	-1	1	21	1047	1067	UUCCACAGCUUUCUUGAACUG	::::::::.: :::::	UCUCACAAGAAAGUUAUGGAA	Cleavage	1
mes-miR396a	MeGATA10	5	-1	1	21	885	905	UUCCACAGCUUUCUUGAACUG	: :.. :::::: ::::.	AUGGUUGUGAAAGCUAUGGAG	Cleavage	1
mes-miR396b	MeGATA23	5	-1	1	21	1062	1082	UUCCACAGCUUUCUUGAACUG	::::::::.: :::::	UCUCACAAGAAAGUUAUGGAA	Cleavage	1
mes-miR396b	MeGATA22	5	-1	1	21	1047	1067	UUCCACAGCUUUCUUGAACUG	::::::::.: :::::	UCUCACAAGAAAGUUAUGGAA	Cleavage	1
mes-miR396b	MeGATA10	5	-1	1	21	885	905	UUCCACAGCUUUCUUGAACUG	: :.. :::::: ::::.	AUGGUUGUGAAAGCUAUGGAG	Cleavage	1
mes-miR396c	MeGATA22	5	-1	1	21	1047	1067	UUCCACAGCUUUCUUGAACUU	::::::::.: :::::	UCUCACAAGAAAGUUAUGGAA	Cleavage	1
mes-miR396c	MeGATA23	5	-1	1	21	1062	1082	UUCCACAGCUUUCUUGAACUU	::::::::.: :::::	UCUCACAAGAAAGUUAUGGAA	Cleavage	1
mes-miR396c	MeGATA10	5	-1	1	21	885	905	UUCCACAGCUUUCUUGAACUU	: : :.. :::::: ::::.	AUGGUUGUGAAAGCUAUGGAG	Cleavage	1
mes-miR396d	MeGATA23	5	-1	1	21	1062	1082	UUCCACAGCUUUCUUGAACUU	::::::::.: :::::	UCUCACAAGAAAGUUAUGGAA	Cleavage	1
mes-miR396d	MeGATA22	5	-1	1	21	1047	1067	UUCCACAGCUUUCUUGAACUU	::::::::.: :::::	UCUCACAAGAAAGUUAUGGAA	Cleavage	1
mes-miR396d	MeGATA10	5	-1	1	21	885	905	UUCCACAGCUUUCUUGAACUU	: : :.. :::::: ::::.	AUGGUUGUGAAAGCUAUGGAG	Cleavage	1
mes-miR396e	MeGATA23	5	-1	1	21	1062	1082	UUCCACAGCUUUCUUGAACUU	::::::::.: :::::	UCUCACAAGAAAGUUAUGGAA	Cleavage	1
mes-miR396e	MeGATA22	5	-1	1	21	1047	1067	UUCCACAGCUUUCUUGAACUU	::::::::.: :::::	UCUCACAAGAAAGUUAUGGAA	Cleavage	1
mes-miR396e	MeGATA10	5	-1	1	21	885	905	UUCCACAGCUUUCUUGAACUU	: : :.. :::::: ::::.	AUGGUUGUGAAAGCUAUGGAG	Cleavage	1
mes-miR396f	MeGATA22	5	-1	1	21	1047	1067	UUCCACAGCUUUCUUGAACUU	::::::::.: :::::	UCUCACAAGAAAGUUAUGGAA	Cleavage	1
mes-miR396f	MeGATA23	5	-1	1	21	1062	1082	UUCCACAGCUUUCUUGAACUU	::::::::.: :::::	UCUCACAAGAAAGUUAUGGAA	Cleavage	1
mes-miR396f	MeGATA10	5	-1	1	21	885	905	UUCCACAGCUUUCUUGAACUU	: : :.. :::::: ::::.	AUGGUUGUGAAAGCUAUGGAG	Cleavage	1
mes-miR397	MeGATA27	5	-1	1	20	918	937	UUUGAGUGCAGCGUUGAUGA	:..::.::: :::.::::	UUGUCGACGAAGCAUUCAAA	Translation	1
